# A surrogate marker of piperaquine-resistant *Plasmodium falciparum* malaria: a phenotype–genotype association study

**DOI:** 10.1016/S1473-3099(16)30415-7

**Published:** 2017-02

**Authors:** Benoit Witkowski, Valentine Duru, Nimol Khim, Leila S Ross, Benjamin Saintpierre, Johann Beghain, Sophy Chy, Saorin Kim, Sopheakvatey Ke, Nimol Kloeung, Rotha Eam, Chanra Khean, Malen Ken, Kaknika Loch, Anthony Bouillon, Anais Domergue, Laurence Ma, Christiane Bouchier, Rithea Leang, Rekol Huy, Grégory Nuel, Jean-Christophe Barale, Eric Legrand, Pascal Ringwald, David A Fidock, Odile Mercereau-Puijalon, Frédéric Ariey, Didier Ménard

**Affiliations:** a Malaria Molecular Epidemiology Unit, Institut Pasteur in Cambodia, Phnom Penh, Cambodia; b Malaria Translational Research Unit, Institut Pasteur, Paris, France; c Institut Pasteur in Cambodia, Phnom Penh, Cambodia; d Department of Parasites and Insect Vectors, Institut Pasteur, Paris, France; e Plate-forme Génomique, Département Génomes et Génétique, Institut Pasteur, Paris, France; f Structural Microbiology Unit, Biology of Malaria Targets Group, Department of Structural Biology and Chemistry and CNRS, UMR3528, Institut Pasteur, Paris, France; g Institut Cochin Inserm U1016, Université Paris-Descartes, Sorbonne Paris Cité, and Laboratoire de Parasitologie-Mycologie, Hôpital Cochin, Paris, France; h National Center for Parasitology, Entomology and Malaria Control, Phnom Penh, Cambodia; i Laboratoire de Mathématiques Appliquées (MAP5) UMR CNRS 8145, Université Paris Descartes, Paris, France; j Department of Microbiology and Immunology and Division of Infectious Diseases, Department of Medicine, Columbia University Medical Center, New York, NY, USA; k Global Malaria Programme, World Health Organization, Geneva, Switzerland

## Abstract

**Background:**

Western Cambodia is the epicentre of *Plasmodium falciparum* multidrug resistance and is facing high rates of dihydroartemisinin–piperaquine treatment failures. Genetic tools to detect the multidrug-resistant parasites are needed. Artemisinin resistance can be tracked using the K13 molecular marker, but no marker exists for piperaquine resistance. We aimed to identify genetic markers of piperaquine resistance and study their association with dihydroartemisinin–piperaquine treatment failures.

**Methods:**

We obtained blood samples from Cambodian patients infected with *P falciparum* and treated with dihydroartemisinin–piperaquine. Patients were followed up for 42 days during the years 2009–15. We established in-vitro and ex-vivo susceptibility profiles for a subset using piperaquine survival assays. We determined whole-genome sequences by Illumina paired-reads sequencing, copy number variations by qPCR, RNA concentrations by qRT-PCR, and protein concentrations by immunoblotting. Fisher’s exact and non-parametric Wilcoxon rank-sum tests were used to identify significant differences in single-nucleotide polymorphisms or copy number variants, respectively, for differential distribution between piperaquine-resistant and piperaquine-sensitive parasite lines.

**Findings:**

Whole-genome exon sequence analysis of 31 culture-adapted parasite lines associated amplification of the *plasmepsin 2*–plasmepsin 3 gene cluster with in-vitro piperaquine resistance. Ex-vivo piperaquine survival assay profiles of 134 isolates correlated with *plasmepsin 2* gene copy number. In 725 patients treated with dihydroartemisinin–piperaquine, multicopy *plasmepsin 2* in the sample collected before treatment was associated with an adjusted hazard ratio (aHR) for treatment failure of 20·4 (95% CI 9·1–45·5, p<0·0001). Multicopy plasmepsin 2 predicted dihydroartemisinin–piperaquine failures with 0·94 (95% CI 0·88–0·98) sensitivity and 0·77 (0·74–0·81) specificity. Analysis of samples collected across the country from 2002 to 2015 showed that the geographical and temporal increase of the proportion of multicopy *plasmepsin 2* parasites was highly correlated with increasing dihydroartemisinin–piperaquine treatment failure rates (r=0·89 [95% CI 0·77–0·95], p<0·0001, Spearman’s coefficient of rank correlation). Dihydroartemisinin–piperaquine efficacy at day 42 fell below 90% when the proportion of multicopy *plasmepsin 2* parasites exceeded 22%.

**Interpretation:**

Piperaquine resistance in Cambodia is strongly associated with amplification of *plasmepsin 2–3*, encoding haemoglobin-digesting proteases, regardless of the location. Multicopy *plasmepsin 2* constitutes a surrogate molecular marker to track piperaquine resistance. A molecular toolkit combining *plasmepsin 2* with *K13* and *mdr1* monitoring should provide timely information for antimalarial treatment and containment policies.

**Funding:**

Institut Pasteur in Cambodia, Institut Pasteur Paris, National Institutes of Health, WHO, Agence Nationale de la Recherche, Investissement d’Avenir programme, Laboratoire d’Excellence Integrative “Biology of Emerging Infectious Diseases”.

## Introduction

Antimalarial efficacy of artemisinin-based combination therapies, the first-line treatment for uncomplicated *Plasmodium falciparum* malaria, relies on both fast-acting artemisinin derivatives and long-lasting partner drugs. Resistance to artemisinin, which is now fixed in western Cambodia and observed across southeast Asia, increases the proportion of parasites surviving a 3 day course of an artemisinin-based combination therapy. Resistance to the partner drug is a greater risk when more parasites survive artemisinin treatment. The reduced efficacy of artemisinin derivatives and partner drugs translates into late treatment failures and prolonged parasite carriage, thereby increasing the transmission potential of drug-resistant infections.

**Research in context**

**Evidence before this study**

We searched PubMed for studies on piperaquine resistance using the term “resistance” in combination with “falciparum” and “piperaquine” on May 19, 2016, without any date or language restrictions, and identified 74 publications. These publications included clinical trials done in 11 countries evaluating the efficacy of dihydroartemisinin–piperaquine for the treatment of uncomplicated *Plasmodium falciparum* malaria (26 reports) or asymptomatic infections (one report) and for intermittent preventive treatment of pregnant women (three reports) or infants (five reports). In all studies, cure rates were above 90%, except studies done in Cambodia after the year 2010, for which cure rates ranging from 85% to 40% were recorded. Overall, 26 publications reported susceptibility of parasites collected in 15 countries, studied using in-vitro or ex-vivo assays. Virtually all isolates tested by standard dose–response susceptibility assays (with parasite quantification based on isotopes, Sybr Green, or HRP2) were susceptible to piperaquine (<100 nmol/L), except those collected in Cambodia after 2010 and samples collected in China before 1998 (when piperaquine monotherapy was intensively used). Piperaquine resistance at present appears confined to Cambodia. Resistance is a major concern because alternative therapeutic options are scarce and the reduced cure rates translate into prolonged parasite carriage and increased transmission potential of resistant parasites. To map the geographical extension of piperaquine resistance and deploy containment measures to prevent its further spread, rapid detection tests are needed but are lacking at present. Potential molecular signatures associated with piperaquine resistance were investigated in 11 studies. The only consistently recorded finding was an increased proportion of single copy *mdr1* parasites in piperaquine-resistant areas. This marker is not informative for piperaquine resistance because wild-type susceptible parasites can also have a single-copy *mdr1* locus.

**Added value of this study**

We identified amplification of the *plasmepsin 2–3* gene cluster encoding proteases involved in haemoglobin degradation as the most significant molecular signature associated with in-vitro resistance to piperaquine assessed using the piperaquine survival assay. Using a large longitudinal collection of samples collected during clinical efficacy studies of dihydroartemisinin–piperaquine done across Cambodia since 2009, we examined 725 *P falciparum* isolates and found that an increased *plasmepsin 2* gene copy number was strongly associated with dihydroartemisinin–piperaquine treatment failures. Patients harbouring multicopy *plasmepsin 2* parasites had a 20 times higher risk of recrudescence during the 42-day post-treatment follow-up (94% sensitivity and 77% specificity). Our retrospective analysis of samples collected in Cambodia during the last decade before and after introduction of dihydroartemisinin–piperaquine as first-line treatment showed that the proportion of multicopy *plasmepsin 2* parasites correlated with the increase of dihydroartemisinin–piperaquine treatment failure rates, from 2009 to 2015 in western Cambodia and during 2014–15 in eastern Cambodia. In areas of artemisinin resistance, the clinical efficacy of dihydroartemisinin–piperaquine at day 42 fell under 90% when the local proportion of multicopy *plasmepsin 2* parasites rose above 22%.

**Implications of all the available evidence**

Dihydroartemisinin–piperaquine failure rates have increased in western Cambodia since 2010 and in eastern Cambodia since 2014. They are caused by parasites that are resistant to both artemisinin and piperaquine. Combined analysis of *K13* polymorphisms and *plasmepsin 2* copy number represents the first informative molecular signature for dihydroartemisinin–piperaquine failures. These molecular markers can now be used to track emergence and dissemination of resistance to artemisinin and piperaquine in field populations, especially in areas where piperaquine is being or will be recommended in combination with artemisinin derivatives as first-line treatment or in preventive treatment for infants or pregnant women, as developed in African settings.

In Cambodia, artesunate–mefloquine was chosen as the first-line drug in 2001. By 2008, the high frequency of treatment failures in western provinces, the epicentre of *P falciparum* multidrug resistance, led to its replacement with dihydroartemisinin–piperaquine in those areas in 2008, and later throughout Cambodia in 2010. In recent years the spread of artemisinin-resistant *P falciparum,* from western Cambodia to neighbouring provinces,^1^, ^2^, ^3^, ^4^, ^5^ has been followed by a substantial increase in dihydroartemisinin–piperaquine failure rates. Failures are estimated to reach 60%,^6^, ^7^, ^8^, ^9^, ^10^ indicating a dramatic expansion of piperaquine resistance. Until now, the detection of piperaquine resistance has been based on logistically demanding 42-day follow-up studies of patients treated with dihydroartemisinin–piperaquine.^11^ The in-vitro piperaquine survival assay (PSA)^7^ has been shown with in-vitro culture-adapted parasites and freshly collected ex-vivo patient isolates to detect piperaquine resistance and treatment failure more reliably than classic dose–response assays.^7^ The in-vitro PSA therefore provides a reliable tool to identify molecular signatures associated with resistance.

Here, we used the phenotypic information from the PSA to identify genetic marker(s) of piperaquine resistance and study their association with dihydroartemisinin–piperaquine treatment failures.

## Methods

### Overview

First, the exomes of culture-adapted artemisinin-resistant Cambodian *P falciparum* lines defined as piperaquine-susceptible or piperaquine-resistant based on their PSA survival rates^7^ were compared for single-nucleotide polymorphisms (SNPs) and copy number variations (CNVs). This process identified an increased copy number of the *plasmepsin 2*–*plasmepsin 3* gene cluster as a putative genetic signature associated with in-vitro piperaquine resistance. Increased *plasmepsin 2* gene copy number was then assessed as a candidate resistance marker in isolates with documented ex-vivo PSA survival rates and in blood samples collected during the years 2009–15 from Cambodian patients treated with dihydroartemisinin–piperaquine and followed up for 42 days. Finally, we investigated the geographical and temporal distribution of multicopy *plasmepsin 2* parasites in the country from 2002 to 2015 and its correlation with dihydroartemisinin–piperaquine treatment failures.

### Study sites and patients

**Figure 1:**
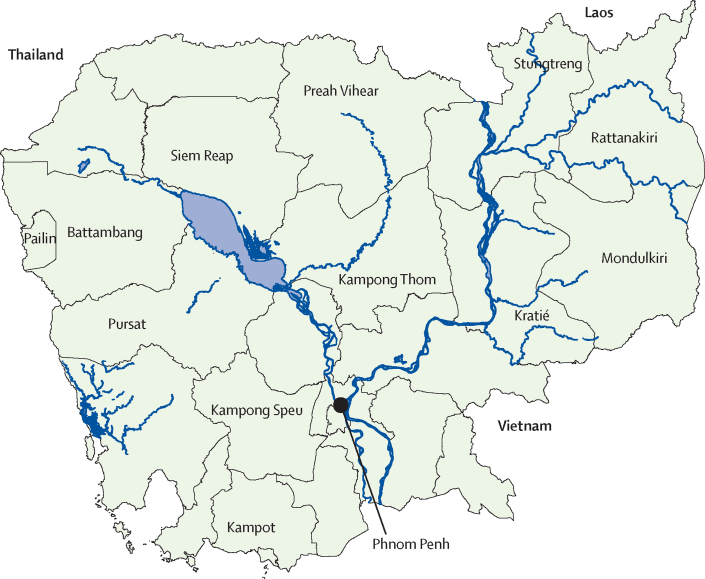
Location of study sites (provinces) where dihydroartemisinin–piperaquine clinical efficacy studies were done in 2009–15 (42-day follow-up)

Patients with *P falciparum* malaria were enrolled in clinical studies done at health centres located across Cambodia during the years 2009–15 (table 1, figure 1). After obtaining written informed consent, patients were treated with dihydroartemisinin–piperaquine (Duo-Cotecxin [dihydroartemisinin 40 mg and piperaquine 320 mg], Zhejiang Holley Nanhu Pharamaceutical Co Ltd, Jiaxing City, Zhejiang Province, China) and followed up for 42 days, as previously described.^7^, ^8^, ^10^ The endpoint to assess the efficacy of dihydroartemisinin–piperaquine was the proportion of PCR-corrected recrudescent *P falciparum* infections at day 42.^11^ All studies were approved by the Ethical Committee for Health Research of the Cambodian Ministry of Health. Clinical trials were registered at the Australian New Zealand Clinical Trials Registry (numbers ACTRN 12615000793516, 12612000184875, 12612000183886, 12612000181808, and 12614000344695).

**Table 1:** Proportion of PCR-corrected *Plasmodium falciparum* recrudescence recorded at day 42 in 2009–15 in 12 provinces across Cambodia in patients treated with a 3-day course of dihydroartemisinin–piperaquine

	**Number of patients treated and followed up (n=725)**	**Number of patients classified as recrudescent (PCR-corrected; n=119)**	**Number of isolates with in-vitro PSA survival data(n=31)**	**Number of isolates with ex-vivo PSA survival data(n=134)**
**2009**				
Pailin	32	3 (9.4%)	0	0
Preah Vihear	30	0	0	0
**2010**				
Pailin	21	4 (19.0%)	0	0
Pursat	32	3 (9.4%)	0	0
Rattanakiri	30	0	0	0
**2011**				
Kratié	51	2 (3.9%)	0	0
Preah Vihear	34	2 (5.9%)	0	0
Pursat	41	7 (17.1%)	0	0
**2012**				
Battambang	39	12 (30.8%)	19	0
Kampong Speu	22	0	4	0
Kampong Thom	38	0	2	0
Pursat	23	2 (8.7%)	6	0
**2013**				
Kampot	17	1 (5.9%)	0	0
Kratié	22	0	0	0
Preah Vihear	16	1 (6.3%)	0	0
Rattanakiri	31	1 (3.2%)	0	0
**2014**				
Mondulkiri	39	4 (10.3%)	0	0
Siemreap	40	25 (62.5%)	0	0
Stungtreng	33	11 (33.3%)	0	0
Rattanakiri	34	5 (14.7%)	0	34
**2015**				
Mondulkiri	16	4 (25.0%)	0	16
Rattanakiri	54	16 (29.6%)	0	54
Siemreap	17	10 (58.8%)	0	17
Stungtreng	13	6 (46.1%)	0	13

Site location and years of collection are provided for isolates with in-vitro and ex-vivo piperaquine survival assay (PSA) profiles (see figure 1 for a map of the study site locations). Data are n or n (%).

### Procedures

Blood samples were collected into acid-citrate-dextrose tubes (Becton-Dickinson, Franklin Lakes, NJ, USA) before treatment and sent to Institut Pasteur in Cambodia within 24 h. A subset of freshly collected samples was used to do the ex-vivo PSA.^7^ All samples were cryopreserved in glycerolyte. Red cell pellets were stored at −20°C for molecular studies. Blood spots were prepared on day 0 and when applicable on the day of recrudescence.

Cryopreserved parasites were culture-adapted as described.^12^ Susceptibility to piperaquine was investigated using in-vitro PSA for culture-adapted parasites and ex-vivo PSA for fresh isolates. Survival rates were assessed microscopically and parasites with a survival rate of at least 10% were considered piperaquine-resistant.^7^ *msp1, msp2*, and *glurp* polymorphisms were determined to distinguish recrudescent from new infections.^13^ Sequencing of the K13-propeller domain was used to *s*creen for artemisinin resistance.^1^ Whole-genome sequencing was done with Illumina paired-reads sequencing.^1^ Data were integrated into the Whole-genome Data Manager database^14^ and exomes of piperaquine-resistant and piperaquine-sensitive lines were compared after excluding low-coverage positions (ie, lower than 25% of the genome-wide mean coverage). Genes from highly variable multigene families (*var, rifin, phist*, and *stevor*) were excluded.^1^ SNPs and CNVs were investigated using PlasmoCNVScan and the Phen2gen software (appendix).^14^ *Plasmepsin 2* and *mdr1* copy number was determined by qPCR (appendix). Steady-state *plasmepsin 2* mRNA concentrations were measured by RT-qPCR (appendix) and plasmepsin 2 protein expression by immunoblotting (appendix).

### Statistical analysis

Data were analysed with MedCalc version 12 (Mariakerke, Belgium). Kruskal-Wallis or Mann-Whitney tests were used for non-parametric comparisons and Student’s *t* test or one-way ANOVA were used for parametric comparisons. For proportions (expressed with percentages and 95% CIs), we used χ^2^ or Fisher’s exact tests. Manhattan plots were generated using the SNPEVG software.^15^ We did an SNP-wise analysis using a homemade script developed by FA and BS and used Fisher’s exact test to identify significant SNP differences between piperaquine-resistant and piperaquine-sensitive parasite lines. We tested CNVs for differential distribution between piperaquine-resistant and piperaquine-sensitive parasite lines using a non-parametric Wilcoxon rank-sum test. The Bonferroni and the Benjamini-Hochberg corrections were used to assess genome-wide significance and adjust p values when statistical tests were done simultaneously on a single dataset (appendix). Relative risks were estimated using the Mantel-Haenszel test. Associations between a cumulative risk of failure at day 42 and molecular signatures associated with piperaquine resistance were assessed by survival analysis. Curves were compared with the Mantel-Haenszel log-rank test. The Cox proportional-hazards regression model was used to assess the association between parasite genotypes (*K13* mutations, *plasmepsin 2,* and *mdr1* copy number), sampling locations, and treatment responses. A linear regression analysis was used to assess the association between the efficacy of dihydroartemisinin–piperaquine and the proportion of parasites with multicopy *plasmepsin 2*. We deemed p values of less than 0·05 as significant.

### Role of the funding source

The funders of this study had no role in study design, data collection, data analysis, data interpretation, writing of the report, and the decision to submit. The corresponding author had full access to all data in the study and final responsibility for the decision to submit for publication.

## Results

From Sept 15, 2009, to Feb 23, 2015, 725 patients were enrolled in clinical studies to assess the efficacy of the standard 3-day dihydroartemisinin–piperaquine treatment. By 2015, the cumulative proportion of *P falciparum* recrudescence at day 42 after PCR correction was 16·4% (119 of 725 patients), ranging from 0% to 62·5% depending on the site and the year of study (table 1, figure 1).

Whole-genome sequences were obtained from 31 artemisinin-resistant (*K13* C580Y mutant) culture-adapted parasite lines collected in Cambodia in 2012, including 23 piperaquine-resistant and eight piperaquine-sensitive lines as defined by their in-vitro PSA survival rates (table 2). We recorded 120 691 exomic (coding sequence) SNPs. Genome-wide association analyses of SNPs identified significant differences between resistant and sensitive lines at two positions located in adjacent genes on chromosome 4: position 896588 of PF3D7_0420000 (encoding a putative zinc-finger protein; p<3·56 × 10^−7^, Fisher’s exact test; p=0·042 after Bonferroni correction) and position 908385 of PF3D7_0420100 (encoding a Rio2 Ser–Thr protein kinase; p<3·56 × 10^−7^, Fisher’s exact test; p=0·042 after Bonferroni correction). However, these positions (and indeed the sequences of both genes) were ambiguous with variable proportions of wild-type and mutant nucleotides, precluding identification of specific resistance-associated mutations, and were not studied further (appendix).

**Table 2:** Details of the 31 K13-C580Y mutant, piperaquine (PPQ)-resistant and PPQ-sensitive culture–adapted parasites analysed by whole-genome sequencing and compared with the 3D7 reference line

	**Year**	**Site location**	**In-vitro PSA survival rate (%)**	In-vitro susceptibility to PPQ*	**DNA expansion type**†
3D7	..	..	0.1%	Sensitive	No amplification
6273	2012	Kampong Speu	0.2%	Sensitive	No amplification
6337	2012	Kampong Speu	0.4%	Sensitive	No amplification
6403	2012	Pursat	0.5%	Sensitive	No amplification
6267	2012	Kampong Speu	0.5%	Sensitive	No amplification
6349	2012	Kampong Thom	0.6%	Sensitive	No amplification
6237	2012	Kampong Thom	0.8%	Sensitive	No amplification
6410	2012	Battambang	6.0%	Sensitive	No amplification
6369	2012	Pursat	6.4%	Sensitive	Type 2
6395	2012	Battambang	19.2%	Resistant	No amplification
6341	2012	Pursat	25.8%	Resistant	Type 2
6280	2012	Battambang	28.9%	Resistant	Type 2
6246	2012	Kampong Speu	36.9%	Resistant	No amplification
6293	2012	Battambang	39.3%	Resistant	Type 2
6391	2012	Battambang	39.4%	Resistant	Type 1
6272	2012	Battambang	40.0%	Resistant	Type 2
6218	2012	Battambang	40.8%	Resistant	Type 1
6302	2012	Battambang	42.5%	Resistant	Type 1
6229	2012	Battambang	46.6%	Resistant	Type 1
6443	2012	Battambang	49.6%	Resistant	Type 1
6430	2012	Battambang	51.3%	Resistant	Type 1
6429	2012	Pursat	51.8%	Resistant	Type 1
6365	2012	Battambang	51.8%	Resistant	Type 2
6394	2012	Battambang	56.7%	Resistant	Type 1
6219	2012	Battambang	58.6%	Resistant	Type 3
6408	2012	Battambang	58.7%	Resistant	Type 3
6224	2012	Pursat	61.4%	Resistant	Type 1
6431	2012	Battambang	61.5%	Resistant	Type 1
6320	2012	Battambang	62.1%	Resistant	Type 1
6261	2012	Pursat	70.5%	Resistant	Type 1
6411	2012	Battambang	71.6%	Resistant	Type 1
6427	2012	Battambang	77.4%	Resistant	Type 3

The last column lists the DNA expansion types recorded in the region of chromosome 14 encoding the plasmepsin 1–4 haemoglobinases.

* Threshold used to define in-vitro susceptibility to PPQ: sensitive if survival rates were less than 10% and resistant if survival rates were 10% or more.

† See appendix for details.

**Figure 2:**
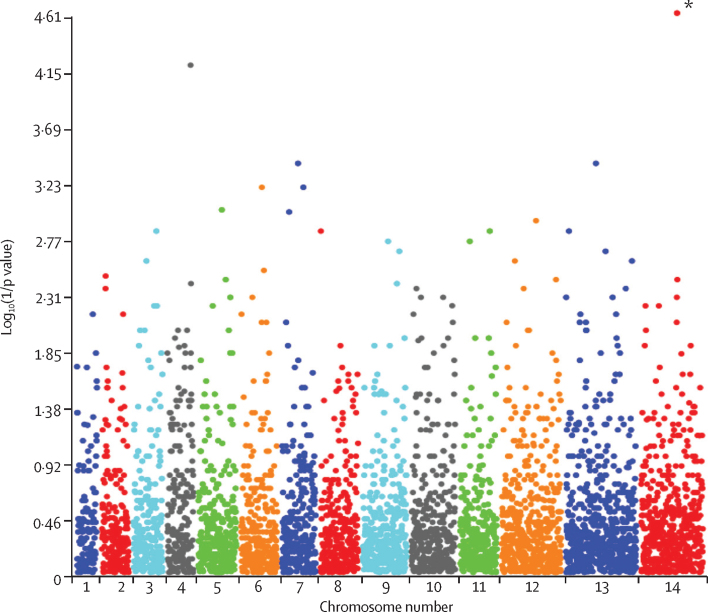
Manhattan plot showing the significance of copy number variations between whole-genome exome sequences of 23 piperaquine-resistant and eight piperaquine-sensitive culture-adapted lines collected in Cambodia in 2012 and phenotyped using the in-vitro piperaquine survival assay Each dot represents a gene in the set of 31 culture-adapted parasites, according to chromosome. The x axis represents genomic location, and the y axis represents the log_10_ transformed Wilcoxon test p values. *Wilcoxon test p=0·139; after Benjamini-Hochberg correction, only two genes, PF3D7_1408000 (*plasmepsin 2*) and PF3D7_1408100 (*plasmepsin 3*) achieved genome-wide significance (p=0·03795).

By contrast, signals of gene amplification were detected in the piperaquine-resistant group for two adjacent genes from the cluster located on chromosome 14 that encode haemoglobin-digesting proteases known as plasmepsins (p=0·03795 Wilcoxon test with Benjamini-Hochberg correction; figure 2, table 3). Irrespective of piperaquine susceptibility, all *plasmepsin 3* sequences were wild type and all *plasmepsin 2* sequences had a Q442H *plasmepsin 2* polymorphism, which has been frequently recorded in reference laboratory lines or wild isolates. The correlation between in-vitro PSA survival rates and *plasmepsin 2–3* copy number was highly significant (*r*=0·83 [95% CI 0·67–0·91], p<0·0001 for *plasmepsin 2* copy number and *r*=0·85 [0·71–0·93], p<0·0001 for *plasmepsin 3* copy number). We recorded three different DNA expansion profiles (table 2, appendix). In-vitro PSA survival rates were significantly lower in parasites harbouring DNA expansion type 2 (n=6, median PSA survival rate 34·1% [IQR 25·8–40·0]) compared with those harbouring DNA expansion type 1 (n=13, median PSA survival rate 51·8% [IQR 45·6–61·7], p=0·006, Mann-Whitney test) or type 3 (n=3, median PSA survival rate 58·7%, p=0·02, Mann-Whitney test).

**Table 3:** List of genes with copy number variation most strongly associated with in-vitro piperaquine resistance expressed by the piperaquine survival assay

	**Gene description**	**Chromosome number**	**Unadjusted Wilcoxon p value**	Bonferroni*	Benjamini-Hochberg*
PF3D7_1408000	*Plasmepsin 2*	14	2.43 × 10^−5^	0.1139	0.03795
PF3D7_1408100	*Plasmepsin 3*, histo-aspartic protease (HAP)	14	2.43 × 10^−5^	0.1139	0.03795
PF3D7_0422000	Steroid dehydrogenase, putative	4	6.54 × 10^−5^	0.306	0.0765
PF3D7_0700800	Pfmc-2TM Maurer’s cleft two transmembrane protein (MC-2TM)	7	4.22 × 10^−4^	1	0.2468
PF3D7_1353100	*Plasmodium* exported protein, unknown function	13	4.22 × 10^−4^	1	0.2468
PF3D7_0713100	Pfmc-2TM Maurer’s cleft two transmembrane protein (MC-2TM)	7	6.65 × 10^−4^	1	0.3112
PF3D7_0605300	Ser–Thr protein kinase (ARK1)	6	6.65 × 10^−4^	1	0.3112
PF3D7_0508400	Transcription factor IIb, putative	5	1.02 × 10^−3^	1	0.4143
PF3D7_0715100	Conserved *Plasmodium* protein, unknown function	7	1.06 × 10^−3^	1	0.4143
PF3D7_1211000	Kinesin-like protein, putative	12	1.25 × 10^−3^	1	0.4208
PF3D7_1304500	Small heat shock protein, putative	13	1.52 × 10^−3^	1	0.4208
PF3D7_1120100	Phosphoglycerate mutase, putative (PGM1)	11	1.52 × 10^−3^	1	0.4208
PF3D7_0315600	Conserved *Plasmodium* protein, unknown function	3	1.52 × 10^−3^	1	0.4208
PF3D7_0800700	Surface-associated interspersed gene 8,3 (SURFIN8,3) (SURF8,3)	8	1.52 × 10^−3^	1	0.4208
PF3D7_1117700	GTP-binding nuclear protein ran/tc4 (RAN)	11	1.85 × 10^−3^	1	0.4572
PF3D7_0909500	Subpellicular microtubule protein 1, putative (SPM1)	9	1.85 × 10^−3^	1	0.4572
PF3D7_1310200	Conserved *Plasmodium* protein, unknown function	13	2.24 × 10^−3^	1	0.4995
PF3D7_0925900	Conserved *Plasmodium* protein, unknown function	9	2.24 × 10^−3^	1	0.4995
PF3D7_0322000	Peptidyl-prolyl *cis-trans* isomerase (CYP19A)	3	2.69 × 10^−3^	1	0.5254

* Based on 4422 genes included in the analysis (total of 4678 screened genes; 256 genes with <500 bp were excluded from the final analysis).

Conversely, a cluster of five genes on chromosome 5 (PF3D7_0531700, PF3D7_0522900, PF3D7_0523000, PF3D7_0523100, and PF3D7_0523200), which included *mdr1*, had increased copy numbers in sensitive lines. *mdr1* was amplified in five of eight piperaquine-sensitive lines but in none of the 23 piperaquine-resistant lines (p=0·015, Wilcoxon test; appendix).

**Figure 3:**
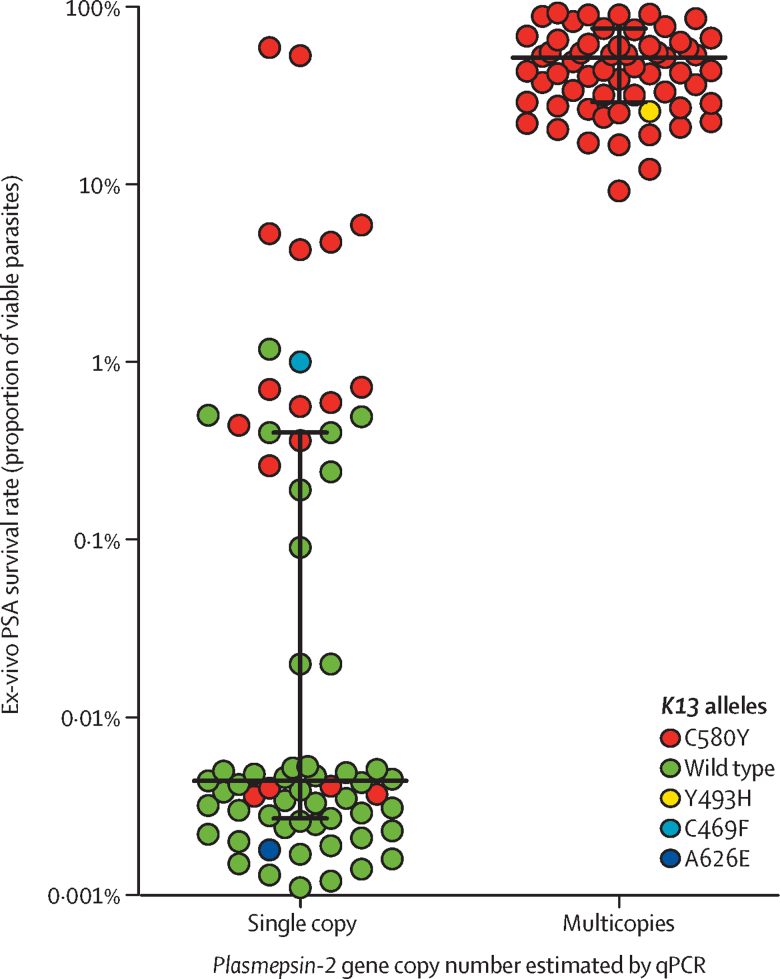
Ex-vivo piperaquine survival assay (PSA) survival rates and single (n=67) and multicopy *plasmepsin 2* (n=67) as estimated by qPCR in isolates collected before dihydroartemisinin–piperaquine (DHA–PPQ) treatment stratified by *K13* genotype Patients were enrolled in clinical studies done in 2014–15 in Mondulkiri, Rattanakiri, Siem Reap, and Stungtreng provinces (see table 1). *K13* polymorphisms were detected in 65 of 69 piperaquine-resistant isolates (64 C580Y, one Y493H) and 17 of 65 piperaquine-susceptible isolates (15 C580Y, one C469F, and one A626E). Three parasite lines with discordant data were recorded: two resistant lines with non-amplified *plasmepsin 2* and *plasmepsin 3* loci (6246 and 6395) and one sensitive line with two *plasmepsin 2* copies (6369; table 2). The ex-vivo PSA survival rate (%) corresponds to the ratio of number of viable parasites in the PPQ-exposed cultures versus the number of viable parasites in the non-exposed culture.

To confirm the association between *plasmepsin* CNV and ex-vivo PSA survival rate, we used *plasmepsin 2* as an amplicon reporter. First, we optimised a qPCR method to assess *plasmepsin 2* gene copy number (appendix). *Plasmepsin 2* copy number detected by qPCR was 100% concordant with the whole-genome sequencing estimates for the 31 culture-adapted parasites (p<0·0001, Fisher’s test). From a set of 134 isolates with known ex-vivo PSA profiles, *plasmepsin 2* was amplified in 67 of 69 piperaquine-resistant parasites (50, 15, and two isolates with two, three, or four *plasmepsin 2* copies, respectively), and zero of 65 piperaquine-susceptible parasites (figure 3). The median ex-vivo PSA survival rate was significantly higher in isolates with at least two *plasmepsin 2* copies compared with those with unamplified *plasmepsin 2* (51·7% [IQR 29·7–75·1] *vs* 0·004% [0·003–0·39]; p<0·0001, Mann-Whitney test). An increased *plasmepsin 2* copy number predicted ex-vivo piperaquine resistance with a sensitivity of 0·97 (95% CI 0·90–0·99) and specificity of 1·00 (0·65–1·00). *K13* polymorphisms were detected in 65 piperaquine-resistant and 17 piperaquine-susceptible isolates (figure 3). Only four of 69 piperaquine-resistant isolates harboured a wild-type *K13* sequence. In a multiple regression analysis, increased *plasmepsin 2* copy number was more strongly associated than *K13* mutations with in-vitro piperaquine resistance (*r*_partial_=0·94, p<0·0001 and *r*_partial_=0·25, p=0·004, respectively).

*Plasmepsin 2* transcript concentrations were 4·1–5·3 times higher in the piperaquine-resistant line ID_6320 compared with the piperaquine-sensitive line ID_6267 at all timepoints of the intra-erythrocytic cycle investigated. Plasmepsin 2 protein concentrations were at least two times higher in piperaquine-resistant parasites ID_6408 compared with the sensitive line ID_6267 (appendix). This finding is consistent with increased protein concentrations in the multicopy *plasmepsin 2* lines studied. However, further work is required to expand this analysis to additional lines.

**Figure 4:**
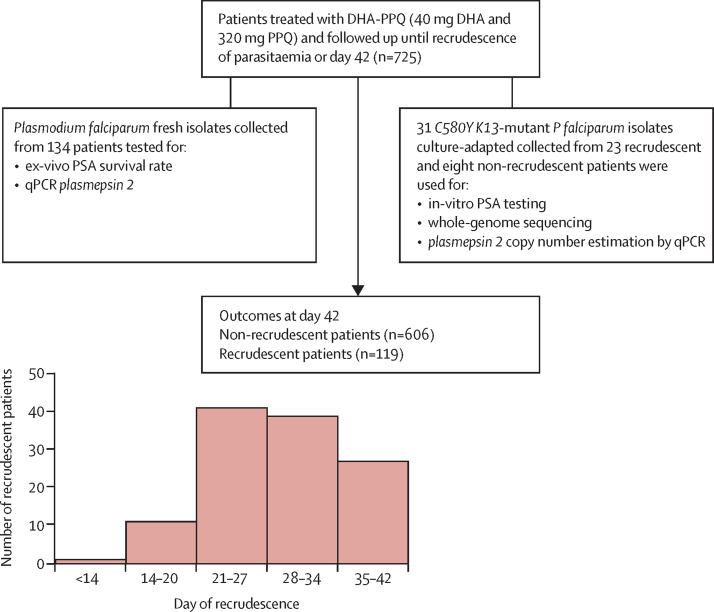
Patients enrolled in clinical studies done in 2009–15 in 12 provinces across Cambodia to assess the efficacy of the 3-day dihydroartemisinin–piperaquine (DHA–PPQ) regimen, and isolates used to detect molecular signatures associated with in-vitro piperaquine survival assay (PSA) resistance and DHA–PPQ clinical failure Supervised DHA–PPQ was given once daily for 3 days (day 0, 24 h, 48 h). Dosing was based on bodyweight: less than 19 kg, 40 mg DHA–320 mg PPQ per day; 19–29 kg, 60 mg DHA–480 mg PPQ per day; 30–39 kg, 80 mg DHA–640 mg PPQ per day; greater than 40 kg, 20 mg DHA–960 mg PPQ per day. For children unable to swallow tablets, DHA–PPQ was dissolved in 5 mL of water. Patients were observed for 1 h post-dosing and were re-dosed with a full or half dose if vomiting occurred within 30 min or between 31 and 60 min, respectively. Those who vomited after the second dose were withdrawn from the study and were given parenteral rescue treatment (intramuscular artemether). Patients with axillary temperatures of 37·5°C were treated with paracetamol. Patients were seen daily to day 3 and then weekly for 6 weeks (day 42) for clinical examinations (axillary temperature, symptom check) and malaria blood films. Home visits were done if patients failed to come back for their follow-up appointments. Withdrawn patients, patients lost to follow-up, and patients classified as reinfected (based on *msp1, msp2,* and *glurp* genotypes) were excluded from the analysis.

**Figure 5:**
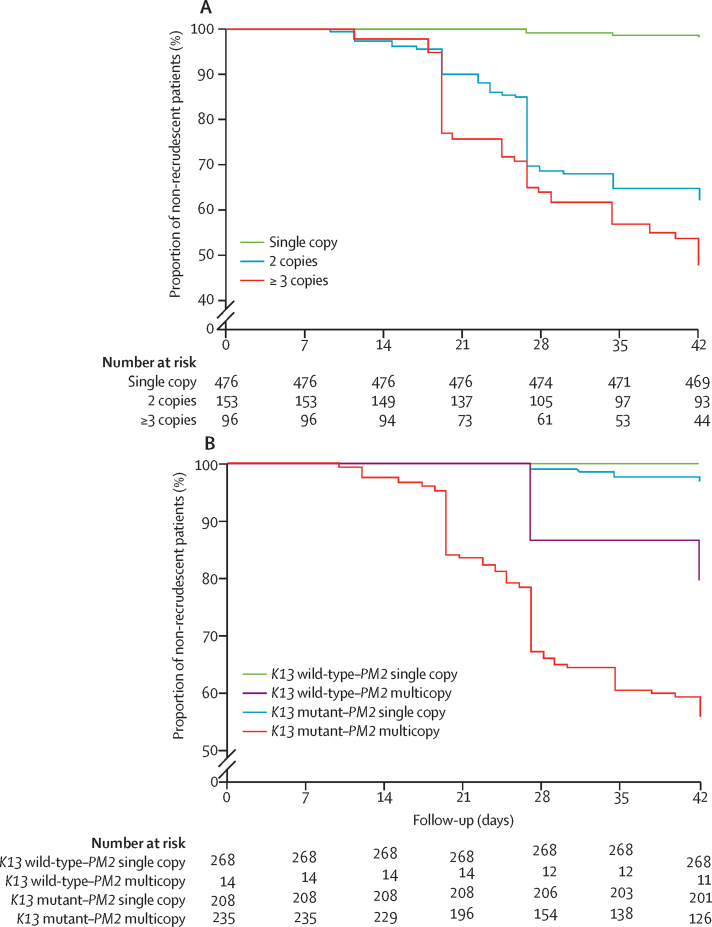
Cumulative proportion of non-recrudescent patients treated with a 3-day course of dihydroartemisinin–piperaquine (A) *Plasmepsin 2* (*PM2*) gene copy number. Log-rank test: p<0·0001 overall; p<0·0001 (hazard ratio [HR] 32·2 [95% CI 17·9–58·0]) for single copy *vs* two copies; p<0·0001 (HR 49·0 [23·0–104·2]) for single copy *vs* three of more copies; p=0·017 (HR 1·53 [1·04–2·25]) for two copies *vs* three or more copies. (B) *PM2* gene copy number and *K13* genotype detected in isolates collected at the time of enrolment, before treatment. Log-rank test: p<0·0001 overall; p<0·0001 for *K13* wild-type–*PM2* single copy *vs K13* wild-type–*PM2* multicopy; p=0·002 for *K13* wild-type–*PM2* single copy *vs K13* mutant–*PM2* single copy; p<0·0001 for *K13* wild-type–*PM2* single copy *vs K13* mutant–*PM2* multicopy; p=0·001 (HR 6·9 [0·5–96·6]) for *K13* wild-type–*PM2* multicopy *vs K13* mutant–*PM2* single copy; p=0·07 (HR 2·6 [1·3–5·5]) for *K13* wild-type–*PM2* multicopy *vs K13* mutant–*PM2* multicopy; p<0·0001 (HR 17·5 [12·2–25·2]) for *K13* mutant–*PM2* single copy *vs K13* mutant–*PM2* multicopy.

We then explored the association between *plasmepsin 2* CNV and dihydroartemisinin–piperaquine treatment outcome in the isolates from 725 patients collected before dihydroartemisinin–piperaquine treatment, of whom 119 experienced recrudescence between day 12 and day 42 (figure 4). *Plasmepsin 2* was unamplified in 476 (65·7%) of 725 isolates, had two copies in 153 (21·1%) of 725 isolates, and three or more copies in 96 (13·2%) of 725 isolates. Only seven (1·5%) of 476 patients with unamplified *plasmepsin 2* parasites had recrudesced by day 42 compared with 112 (45·0%) of 249 patients infected with multicopy *plasmepsin 2* parasites (relative risk [RR] 22·8 [95% CI 10·7–48·6], p<0·0001). Recrudescence was more frequent for isolates with three or more *plasmepsin 2* copies compared with those with two copies (52 [54·2%] of 96 *vs* 60 [39·2%] of 153, p=0·02). The cumulative incidence of dihydroartemisinin–piperaquine treatment failure increased with increasing *plasmepsin 2* gene copies: unamplified versus two copies, hazard ratio (HR) 32·2 (95% CI 17·9–58·0), p<0·0001; unamplified versus three copies, HR 49·0 (23·0–104·2), p<0·0001; or two copies versus three or more copies, HR 1·53 (1·04–2·25), p=0·017 (figure 5A). The mean time to recrudescence decreased with increasing *plasmepsin 2* copy number: 41·9 days (95% CI 41·8–42·0) for patients with unamplified *plasmepsin 2*, 36·0 days (34·6–37·4) for those with two copies, or 34·0 days (32·1–35·0) for those with three or more copies. Increased *plasmepsin 2* copy number predicted dihydroartemisinin–piperaquine treatment failures with a sensitivity of 0·94 (95% CI 0·88–0·98) and a specificity of 0·77 (0·74–0·81). The AUC (area under the ROC curve) was 0·86 (95% CI 0·83–0·88), significantly different from 0·5, the reference value of the null hypothesis (p>0·0001).

Among the 725 patients treated with dihydroartemisinin–piperaquine, *K13* mutants were detected in 443 (61·1%) of 725 day 0 isolates (figure 5B). Of these, 116 (26·2%) of 443 were from patients who failed dihydroartemisinin–piperaquine treatment by day 42 compared with three (1·1%) of 282 from patients harbouring *K13* wild-type parasites (RR 24·6 [95% CI 7·9–76·7], p<0·0001). A single *mdr1* gene copy was detected in 610 (84·1%) of 725 day 0 isolates. Dihydroartemisinin–piperaquine failures were recorded in 112 (18·4%) of 610 patients infected with parasites harbouring a single *mdr1* copy and seven (6·1%) of 115 patients infected with multicopy *mdr1* parasites (RR 3·0 [95% CI 1·4–6·3], p=0·003). The cumulative incidence of dihydroartemisinin–piperaquine treatment failure did not increase with increasing age (stratified in three classes: 0–15 years, 16–30 years, and >30 years; p=0·1809, log-rank test) or with increasing parasite numbers in isolates collected before dihydroartemisinin–piperaquine treatment (stratified in four classes: <5000 parasites per μL, 5001–20 000 parasites per μL, 20 001–50 000 parasites per μL, and >50 000 parasites per μL; p=0·4612, log-rank test).

After controlling for *K13* and *mdr1* genotypes in a Cox proportional-hazards regression model, *plasmepsin 2* copy number (any increase compared with non-amplification) was the most significant molecular signature associated with dihydroartemisinin–piperaquine treatment failure (adjusted HR [aHR] 20·4 [95% CI 9·1–45·5], p<0·0001), followed by *K13* mutation (aHR 5·5 [1·7–18·3], p=0·005), then *mdr1* single copy (aHR 2·05 [0·95–4·42], p=0·06). The cumulative incidence of dihydroartemisinin–piperaquine treatment failure among patients harbouring artemisinin-resistant parasites (ie, an artemisinin resistance-associated *K13* mutation) increased significantly with *plasmepsin 2* copy number (unamplified *vs* two or more copies, seven [3·3%] of 208 *vs* 109 [46·4%] of 235; HR 17·5 [95% CI 12·2–25·2]).

CNVs in *plasmepsin 2* were investigated in 1252 samples collected across Cambodia from 2002 to 2015 (ie, before and after the introduction of dihydroartemisinin–piperaquine). This sample included 527 archived isolates in addition to the 725 studied above (appendix). A longitudinal sampling was done in Pailin (western Cambodia) and Rattanakiri (eastern Cambodia), where dihydroartemisinin–piperaquine was introduced in 2008 and 2010, respectively. In Pailin, the proportion of multicopy *plasmepsin 2* parasites increased from 27·9% (19 of 68) in 2008–09 to 91·2% (52 of 57) in 2014–15. In Rattanakiri, multicopy *plasmepsin 2* parasites were infrequent until 2012–13 (3·2% [one of 31]) but increased to 45·5% (40 of 88) in 2014–15 (appendix). A steady increase of multicopy *plasmepsin 2* parasites after introduction of dihydroartemisinin–piperaquine was recorded in other provinces as well (Preah Vihear, Pursat; appendix).

In the 12 sites where dihydroartemisinin–piperaquine efficacy studies were done in 2009–15, the proportion of multicopy *plasmepsin 2* isolates was negatively correlated with day 42 cure rates (*r*=0·89 [95% CI 0·77–0·95], p<0·0001; appendix). A Cox regression model showed that the risk of recrudescence following a dihydroartemisinin–piperaquine 3-day course was significantly associated (p<2 × 10^−16^) with the presence of multicopy *plasmepsin 2* parasites on day 0 irrespective of the site of enrolment (appendix). A linear regression model showed that the clinical efficacy of dihydroartemisinin–piperaquine at day 42 fell below 90% when the proportion of multicopy *plasmepsin 2* parasites on K13-mutant genetic background rose above 22%.

## Discussion

Following reports of increasing failure of artesunate–mefloquine in western Cambodia, dihydroartemisinin–piperaquine was adopted in 2008 in the western provinces and implemented nationwide in 2010. Resistance to this combination has recently accelerated to levels that render it widely ineffective.^1^ The dearth of alternatives creates a perilous situation whereby these multidrug-resistant infections might become untreatable and spread to other regions with endemic malaria.

The strategy used here to search for genetic associations with piperaquine resistance relied on genome-wide sequence comparisons of a set of artemisinin-resistant parasite lines collected in Cambodia in 2012, all harbouring the C580Y artemisinin resistance mutation and presenting in-vitro PSA survival rates indicative of piperaquine resistance or susceptibility. We reasoned that such a focused sampling in a geographically restricted population would reduce the genetic noise of artemisinin responses and population structure. Results identified amplification of the *plasmepsin 2–3* cluster as a putative genetic event associated with piperaquine resistance. To confirm this association across the country, we focused on *plasmepsin 2*, located in the centre of the amplicon. *Plasmepsin 2* amplification strongly correlated with ex-vivo PSA survival rates irrespective of artemisinin susceptibility and was highly predictive of dihydroartemisinin–piperaquine failures in all geographical areas of Cambodia. *Plasmepsin 2* amplification thus represents an informative marker for piperaquine resistance.

The strong association between *K13* polymorphisms and *plasmepsin 2* amplification in the Cambodian parasites studied herein most likely reflects the history of drug selection in Cambodia. The proportion of isolates with different *K13*–*plasmepsin 2* combinations (appendix) is consistent with a stepwise selection first for artemisinin resistance then for piperaquine resistance. This scenario is in line with the delayed appearance of multicopy *plasmepsin 2* parasites in eastern provinces where the emergence of artemisinin resistance was delayed compared with western provinces (appendix). Dihydroartemisinin–piperaquine treatment failures were rare in eastern Cambodia by 2013, confirming observations by others^6^ but increased steadily in 2014 to reach a high frequency by 2015. Most treatment failures had a single gene copy of *mdr1* (112 [94·1%] of 119), confirming earlier reports of failures.^6^, ^7^, ^8^, ^9^ The presence of single copy *mdr1* is consistent with data reported for in-vitro-selected piperaquine-resistant Dd2 parasites^16^ and analysis of field samples from Cambodia, suggesting opposing resistance mechanisms against these molecules.^17^ We did not observe the *crt* C101F mutation recorded in a piperaquine-pressured parasite line selected in vitro. Thus, our data show that although the most informative marker for piperaquine resistance is *plasmepsin 2* copy number, mutation of *K13* alongside a single *mdr1* gene copy contributes to the dihydroartemisinin–piperaquine failure phenotype. This finding does not exclude the possibility that additional genes contribute to piperaquine resistance. In particular, the significance of the mutations observed for PF3D7_0420000 and PF3D7_0420100 is unclear. Whether the notable sequence heterogeneity of both genes reflected ongoing purifying selection associated with piperaquine resistance or loss of mefloquine resistance is uncertain. Analysis of a larger number of isolates with documented phenotypes for both mefloquine and piperaquine is needed to address this question.

Drug-selected gene amplification is a well-known phenomenon in malaria parasites.^18^, ^19^, ^20^, ^21^ The size of the amplicons on chromosome 14 varied depending on the isolate, as reported for *mdr1*.^22^ Gene amplification, which is more frequent than point mutation in *P falciparum* parasites,^22^ is consistent with the remarkably rapid rise and spread of piperaquine resistance in Cambodia. Conversely, *mdr1* de-amplification, consistent with regained susceptibility to mefloquine, occurred in Cambodia in recent years,^6^, ^8^, ^23^ and, as shown here, is associated with the emergence of piperaquine-resistant strains.

Plasmepsins are expressed during the intra-erythrocytic asexual blood stage cycle and by sexual stage gametocytes that can be transmitted to the mosquito vector. All four plasmepsins are located in the digestive vacuole of intra-erythrocytic developmental forms where they engage in different steps of haemoglobin degradation. Studies of parasites disrupted in the *plasmepsin* genes pointed to redundancy in the haemoglobin degradation machinery.^24^ To our knowledge, there are no reported studies about the consequences of overexpressing these proteases. We show here that *plasmepsin 2* amplification is associated with a notable increase of steady-state mRNA and protein concentrations in two culture-adapted isolates. This observation needs to be confirmed with additional isolates. A reasonable hypothesis is that the amplification of plasmepsins overcomes the inhibitory effect of piperaquine on haemoglobin degradation and haem detoxification, possibly by reducing concentrations of reactive haem species that are preferred substrates for piperaquine binding. Piperaquine-treated trophozoites have been shown to possess large digestive vacuoles containing membrane-bound packets of undigested haemoglobin.^25^ The observation that piperaquine-resistant parasites have a single *mdr1* copy is consistent with this scenario, since maintenance of a single *mdr1* copy (or reversion to a single copy) might avoid importing excessive amounts of piperaquine into the digestive vacuole (appendix).^26^, ^27^

We note that the association of piperaquine resistance with amplification of the *plasmepsin 2–3* cluster on chromosome 14 is not proof of causality. The structured populations of *P falciparum* parasites in Cambodia^28^ might confound the robustness of the association and additional loci might also contribute to piperaquine resistance. The present findings should be complemented with laboratory investigations of the cellular consequences of this amplification on the parasite response to piperaquine and on parasite fitness and transmissibility. Nonetheless, our data are timely in providing a molecular tool that predicts the appearance of piperaquine resistance in endemic settings.

Piperaquine is a well-tolerated partner drug used in combination with artemisinin derivatives or the ozonide compound arterolane (OZ277).^29^ The mechanism of piperaquine resistance in the specific context of Cambodia, where artemisinin resistance is nearly fixed and drug pressure is high, might not extrapolate to areas where artemisinin resistance has not yet been documented. Nevertheless, we propose to extend the assessment of *plasmepsin 2* gene copy number to areas where piperaquine is being used in artemisinin-based combination therapies at a very large scale, and to combine this assay with *K13* sequencing to localise areas of parasite resistance to both components. In Cambodia, where the rapid failure of first-line artemisinin-based combination therapies is jeopardising elimination efforts and accelerating the emergence and spread of resistance, the opposing susceptibility between mefloquine and piperaquine could be used to implement new strategies based on artemisinin-based combination drug rotation, sequential administration, or triple combinations including both artemisinin-based combination partner drugs. Although challenging to implement, these alternative strategies will help to ensure long-term efficacy of antimalarials to reach the elimination goal.
