# Corrections

**DOI:** 10.1016/S1473-3099(18)30396-7

**Published:** 2018-08

*Witkowski B, Duru V, Khim N, et al. A surrogate marker of piperaquine-resistant Plasmodium falciparum malaria: a phenotype–genotype association study.* Lancet Infect Dis *2017; **17**: 174–83*—In this Article, the copyright line should have been “© 2016 The Author(s). Published by Elsevier Ltd. This is an Open Access article under the CC BY 4.0 license.” This correction has been made to the online version as of June 12, 2018.

