# Supplementary material for: A surrogate marker of piperaquine-resistant Plasmodium falciparum malaria: a phenotype–genotype association study
Source: Lancet Infect Dis. 2017 Feb;17(2):174–83. doi: 10.1016/S1473-3099(16)30415-7 (PMC5266792; doi:10.1016/S1473-3099(16)30415-7)
Supplement: Supplementary appendix [file mmc1.pdf]

THE LANCET Infectious Diseases

Supplementary webappendix

This webappendix formed part of the original submission and has been peer reviewed. We post it as supplied by the authors.

Supplement to: Witkowski B, Duru V, Khim N, et al. A surrogate marker of piperaquine-resistant *Plasmodium falciparum* malaria: a phenotype–genotype association study. *Lancet Infect Dis* 2016; published online Nov 3. http://dx.doi.org/10.1016/S1473-3099(16)30415-7.

APPENDIX MATERIAL

A surrogate marker of piperaquine-resistant *Plasmodium falciparum* malaria

Benoit Witkowski*, Valentine Duru*, Nimol Khim, Leila S Ross, Benjamin Saintpierre, Johann Beghain, Sophy Chy, Saorin Kim, Sopheakvatey Ke, Nimol Kloeung, Rotha Eam, Chanra Khean, Malen Ken, Kaknika Loch, Anthony Bouillon, Anais Domergue, Laurence Ma, Christiane Bouchier, Rithea Leang, Rekol Huy, Grégory Nuel, Jean-Christophe Barale, Eric Legrand, Pascal Ringwald, David A Fidock, Odile Mercereau-Puijalon, Frédéric Ariey, Didier Ménar
*Contributed equally

CONTENTS

Appendix 1: Detailed methods: Piperaquine Survival Assays (in-vitro and ex-vivo), DNA, RNA and protein extraction, Whole-genome sequencing and immunoblotting.

Appendix 2: *PfPM2 and Pfmdr1* copy number determination, with listing of primers, protocols and PCR amplification efficiencies.

Appendix 3: *PfPM2* mRNA expression profile, with listing of primers, protocols and RT-qPCR amplification efficiencies.

Appendix 4: Panel A. Manhattan plot showing the significance of single nucleotide polymorphisms (SNPs) between whole-genome exome sequences of 23 piperaquine-resistant and 8 piperaquine-sensitive culture-adapted lines phenotyped using in-vitro PSA. **Panel B.** List of the positions with variable proportions of wild type and mutant nucleotides of PF3D7_0420000 (encoding a putative zinc finger protein) and PF3D7_0420100 (encoding the Rio2 serine/threonine protein kinase) sequences of 23 piperaquine-resistant and 8 piperaquine-sensitive culture-adapted lines phenotyped using in-vitro PSA.

Appendix 5: Profiles of DNA expansion in the region of genes encoding proteins involved in hemoglobin-degrading activities positively associated with *in vitro* piperaquine resistance and methodology developed to confirm the four DNA expansion profiles.

Appendix 6: Distribution of the Wilcoxon Rank-Sum test p-values ranking the significance of CNVs of the 4,616 genes screened between whole-genome exome sequences of 23 piperaquine-resistant and 8 piperaquine-sensitive culture-adapted lines phenotyped using in-vitro PSA.

Appendix 7: Panel A. *PfPM2* mRNA transcript levels, relative to *Pfserine-tRNA ligase* mRNA in early ring stages, late ring stages, early trophozoite stages and mature trophozoites of in-vitro culture-adapted piperaquine-resistant and piperaquine-sensitive parasite lines.
Panel B. PfPM2 expression in *Plasmodium falciparum* synchronized trophozoite-stage of culture-adapted piperaquine-resistant and piperaquine-sensitive parasite lines detected by western immunoblot.

Appendix 8: Spatio-temporal increase in frequency of parasites with multicopy *PfPM2* in Cambodia between 2002 and 2015. **Panel A.** Increase in frequency of parasites with multicopy *PfPM2* in Pailin (red histogram) and Rattanakiri (black histogram) between 2002 and 2015. **Panel B.** Dynamic of the spread of multicopy *PfPM2* parasites in 10 Cambodian provinces from 2002 to 2015

Appendix 9: Correlation between the proportion of parasites with multicopy *PfPM2* and DHA-PPQ treatment failure rates recorded in 12 sites across Cambodia from 2009 to 2015.

Appendix 10: Proportions of isolates with different genetic background (K13 wild-type/*PfPM2* single copy, K13 mutant/*PfPM2* single copy, K13 mutant/*PfPM2* multicopy and K13 wild-type/*PfPM2* multicopy).

Appendix 11: Hypothesis supporting the mechanisms of resistance of *P. falciparum* parasites to PPQ through the amplification of *PfPM2* and *PfPM3* genes, and the deamplification of the *Pfmdr1* gene in Cambodia.

Appendix 1: Detailed methods: Piperaquine Survival Assays (in-vitro and ex-vivo), DNA, RNA and protein extraction, Whole-genome sequencing and immunoblotting.

Piperaquine Survival assays (PSA). In-vitro susceptibility to PPQ was investigated using the in-vitro or the ex-vivo PSA, which is based on exposing very early ring-stage parasites to 200 nM PPQ for 48 hours, washing away the drug, and assessing parasite growth after a further 24 hours of culture. Survival rates at the 72 hour time point were assessed microscopically by counting the proportion of viable parasites in exposed and non-exposed cultures that developed into second-generation rings or trophozoites with normal morphology. Parasites with a survival rate ≥10% were considered PPQ-resistant.[5]

DNA, RNA and protein extraction. Parasite DNA was extracted from blood spots with Instagen matrix (Bio-Rad, Marnes-la-Coquette, France), and from whole blood or cultured parasites with QIAamp DNA Blood MiniKit (Qiagen, Valencia, CA). Total RNA was isolated from cultured parasites using a Trizol reagent-based protocol (Life Technologies, Courtaboeuf, France) and purified with the RNeasy Mini Kit (Qiagen, Valencia, CA). Samples were DNase-treated (Life Technologies) to remove any contaminating genomic DNA. Proteins were extracted from cultured parasites that had been lysed with 0.15% saponin in PBS. The parasite pellet was washed four times with PBS, resuspended at 400,000 parasites per µL of PBS with 1x protease inhibitor cocktail (Sigma-Aldrich, St. Louis, MO, USA) and lysed with a BioRuptor Twin (10 cycles of 10 seconds each, low intensity).

Whole-genome sequencing. Image analysis, base calling and error estimation used the Illumina Analysis Pipeline version 1.7. Raw sequence files were filtered using Fqquality tool. Trimmed reads from controlled Fastq files were mapped onto the *P. falciparum* 3D7 reference genome with the Burrows-Wheeler Alignment (BWA), generating BAM files (binary files of tab-delimited format SAM). Samtools was used to prepare pileup files, which were formatted using in-house software to integrate the data into the Whole-genome Data Manager (WDM) database.[15] Exomes of PPQ-resistant and -sensitive culture-adapted lines were compared after excluding positions with coverage lower than 25% of the genome-wide mean. SNPs were explored after excluding genes from highly variable multi-gene families (*var, rifin, phist* and *stevor*), as described.[1]

Analysis of Single Nucleotide Polymorphisms (SNPs)

The analysis was based on the data generated by the samtools Pileup tools (based on sam file), which incorporates short indels information by correcting the effect of flanking tandem repeats. For each position, any information present in at least 20% of the sequences of that position (≥20% coverage) was considered. Sequence polymorphism was presented using uppercase IUPAC codes in case of base substitutions and a lowercase IUPAC code in case of short indels. This allowed included in a single analysis both types of mutations (30 different codes). The IUPAC Nucleotide code is: A: Adenine, C: Cytosine, G: Guanine, T: Thymine, R: A or G, Y: C or T, S: G or C, W: A or T, K: G or T, M: A or C, B: C or G or T, D: A or G or T, H: A or C or T, V: A or C or G, N: any base. If an indel is observed at a given position, letters are shown in lowercase.

For each nucleotide position, a contingency table was made: the number of columns was variable according to the number of variants observed whereas the line number was constant (sensitive and resistant according to the PSA value). 3D7 was included in the piperaquine-sensitive set. The Bonferroni correction was used to evaluate the significance of the observed polymorphism.

Analysis of Copy Number Variations (CNVs)
Copy Number Variations (CNVs) (after excluding indels) were investigated using PlasmoCNVScan and Phen2gen software's.[15] PlasmoCNVScan is a C/C++ software that normalizes read depth (coverage) across the entire genome, thus by-passing the risk that the sequencing process is not uniform (i.e., the number of reads mapped to a region is assumed to follow a Poisson distribution and is proportional to the number of copies) and the need of reads mapped against a well-annotated reference genome. The underlying concept of ReadDepth-based methods is that the depth of coverage in a genomic segment is correlated with the copy number of the segment. Specifically, we first computed the average frequency for each motif (6-mer) across the whole exome, generating the genome coverage for each motif. We next recorded the local coverage for a motif at each position (extracted from the pileup file). Then, for each gene, we used a sliding window, computed the ratio between observed coverage and genome coverage for each position and calculated the average coverage for that gene. In the final analysis, genes with nucleotide sequence lengths less than 500 bp were excluded. We considered CNV as a continuous variable and used the Wilcoxon test to compare CNVs of PPQ-resistant and -sensitive parasite lines. CNVs were also classified according to their Wilcoxon rank-sum values. The Bonferroni and Benjamini-Hochberg corrections were as used to evaluate the significance of the CNVs.

Immunoblotting. Parasite lysates (synchronized trophozoite-stage cultures; 24-30 hours post invasion), were mixed with complete Laemmli buffer and boiled for 10 minutes at 95C. Samples were run on a 10% Tris-Gly-SDS precast gel (BioRad) at 120V for 2 h with a Precision Kaleidoscope protein marker (Biorad). The gel contents were transferred to a nitrocellulose membrane (315 mA 90 minutes). Membranes were blocked with 2% nonfat dry milk and 1% BSA in TBS for 90 minutes at room temperature. Membranes were probed with antibody diluted in the blocking buffer at 4°C overnight. Dilutions used were 1:2,000 for anti-Plasmepsin2 (gift from Dan Goldberg) and 1:5000 for anti-beta actin (NovusBio). Membranes were washed in TBST, then probed with the appropriate 1:10,000 secondary antibody in blocking buffer for one hour at room temperature. Membranes were washed with TBS, then treated with ECL (Pierce) and exposed to film.

Appendix 2. *PfPM2 and Pfmdr1* copy number determination, with listing of primers, protocols and PCR amplification efficiencies.

PfPM2 (PF3D7_1408000) and *Pfmdr1* (PF3D7_0523000) copy numbers were measured by qPCR using a CFX96 real-time PCR machine (Bio-Rad). As a reference, we used the single copy β-*tubulin* (PF3D7_1008700) gene. Listing of primers, protocols and PCR amplification efficiencies are provided in the table below.

qPCR	Primer Sequence	Sequences	Tm (°C)	Product size (bp)	Range of Melt T°C
PfPM2	PfPM2_CN_F	5'-TGGTGATGCAGAAGTTGGAG-3'	59.8	79	76.8 -77.2
	PfPM2_CN _R	5'-TGGGACCCATAAATTAGCAGA-3'	59.4		
	Pf β-tubulin_CN_F	5'-TGATGTGCGCAAGTGATCC-3'	61.9	79	79.0 - 79.2
	Pf β-tubulin_CN_R	5'-TCCTTTGTGGACATTCTTCCTC-3'	60.5		
Pfmdr1	Pfmdr1_CN _F	5'-TGCATCTATAAAACGATCAGACAAA-3'	60.0	87	77.8 - 78.0
	Pfmdr1_CN_R	5'-TCGTGTGTTCCATGTGACTGT-3'	60.0		
	Pf β-tubulin_CN_F	5'-TGATGTGCGCAAGTGATCC-3'	61.9	79	79.0 - 79.2
	Pf β-tubulin_CN_R	5'-TCCTTTGTGGACATTCTTCCTC-3'	60.5		

PfPM2 copy number.

Quantitative PCR (qPCR) was carried out in 20 µl volumes in a 96-well plate containing 5X HOT FIREPol EvaGreen qPCR Mix ROX (Solis BioDyne, Estonia), 0.25 µM of each forward and reverse primer and 4 µl of template DNA. Final $MgCl_2$ concentrations were 2.5 mM and 4 mM for *PfPM2* and *Pf*β-*tubulin*, respectively. Amplifications were performed under the following conditions: 95°C for 15min, followed by 45 cycles of 95°C for 15s, 58°C for 20s, and 72°C for 20s.

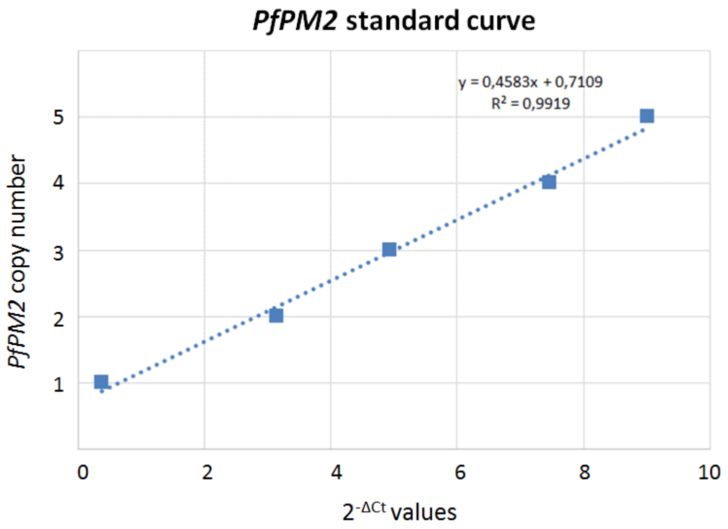

PfPM2 copy number of each sample was measured in triplicate relative to a standard curve using five standards of mixed synthetic gene fragments (Eurofins Genomics, Ebersberg, Germany). The lengths of the synthetic fragments for PfPM2 and Pfβ-tubulin, including gene locations are for *PfPM2* (PF3D7_1408000, from position 367 to 560, 193 bp): 367-aggtagttcaaatgataatatcgaattagtagatttccaaaatataatgtttta**tggtgatgcagaagttggagataaccaacaaccatttacatttattcttgatacaggatctgctaatttatgggtccca**agtgttaaatgtacaactgcaggatgtttaactaaacatctatatgattcatctaaatc-560; and for *Pf*β-*tubulin* (PF3D7_1008700): from positions 1183 to 1391 (208 bp): 1183-tcaacaatacagagccttaactgtgccggagttaacacaacaaatgttcgacgcaaaaaaata**tgatgtgcgcaagtgatccaagacatggaagatatttaacggcatgtgctatgtttagaggaagaatgtccacaaagga**agttgacgaacaaatgttaaacgttcaaaataaaaactcatcttattttgtcgaatggattcctcac-1391 (shown in bold font, the qPCR amplified portion).

The five standards of mixed synthetic gene fragments were: standard 1 (1:1 molar ratio of *PfPM2* & β-tubulin), standard 2 (2:1 molar ratio of *PfPM2* & β-tubulin), standard 3 (3:1 molar ratio of *PfPM2* & β-tubulin), standard 4 (4:1 molar ratio of *PfPM2* & *β-tubulin*) and standard 5 (5:1 molar ratio of *PfPM2* & *β-tubulin*).

The 3D7 (Africa) line was included in each run as control (one copy of *PfPM2*). *PfPM2* copy number was calculated by the $2^{-\Delta Ct}$ method ($\Delta C_t = C_{t\ PfPM2} - C_{t\ Pf\beta\text{-}tubulin}$ where C_t is the threshold cycle) and deduced from the standard curve. A *PfPM2* copy number >1.6 was defined as an amplification of the gene. Amplification efficiencies of the *PfPM2* and the *Pfβ-tubulin* genes, measured using ten-fold dilutions of 3D7 DNA, were similar (99% and 95%, respectively).

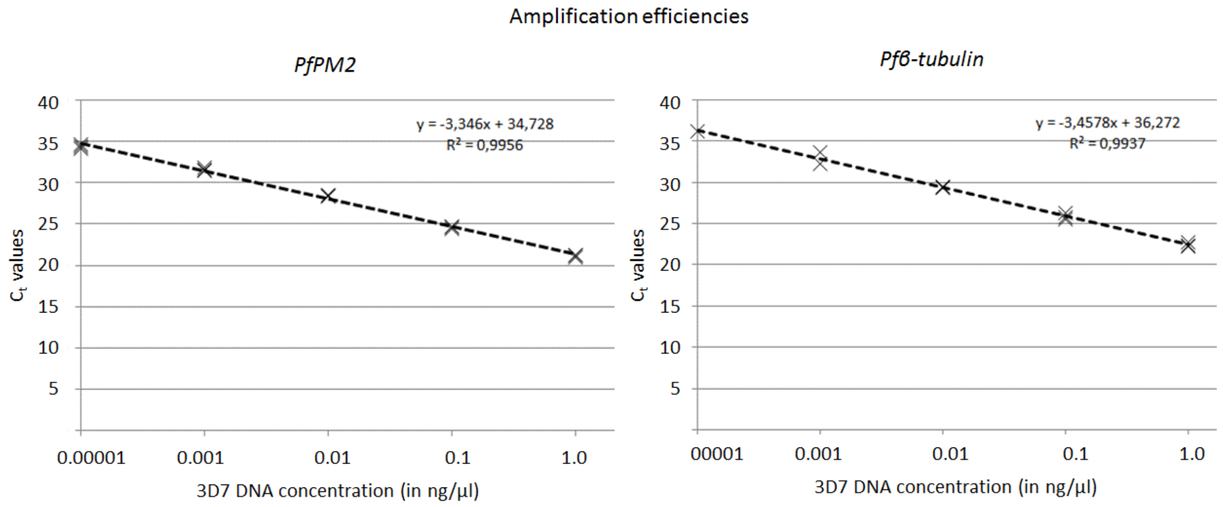

***Pfmdr1* copy number.**
qPCRs were carried out in 25 µl volumes in a 96-well plate containing 5X HOT FIREPol EvaGreen qPCR Mix Plus (Solis BioDyne, Estonia), 0.3 µM concentrations of each forward and reverse primers, and 4 µl of template DNA. Amplifications were performed under the following conditions: 94°C for 15min, followed by 40 cycles of 94°C for 15s, 58°C for 20s, and 72°C for 20s. For each run, the *Pfmdr1* copy number of each sample was measured in triplicate relative to a standard curve using four standards of mixed synthetic gene fragments (Eurofins Genomics, Ebersberg, Germany). The lengths of the synthetic fragments for *Pfmdr1* (F3D7_0523000), including gene location are for *PfPM2* (PF3D7_1408000), from position 3981 to 4260 (204 bp): 3981- ctattgtagatattaaagataaagctgacaaaactattattactattgcccacagaat**tgcatctata aaacgatcagacaaaattgtggtatttaataaccctgatcgaaatggaacctttgtacagtcacatggaacacacga**tgaattattatc agcacaagatggaatatataaaaaatatgtaaaattagctaaatga-4260 (shown in bold font, the qPCR amplified portion).

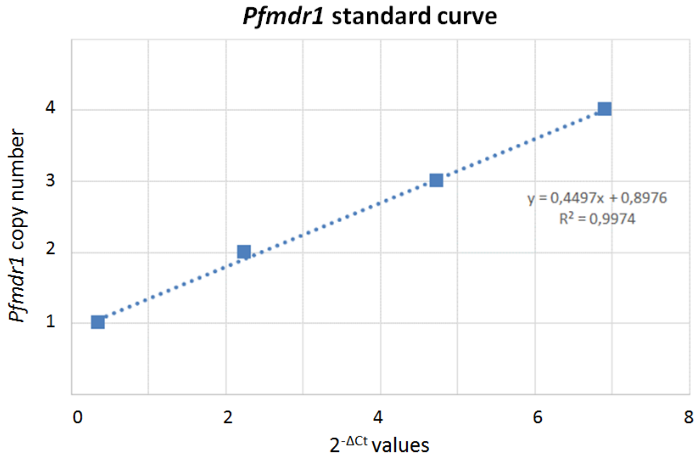

The four standards of mixed synthetic gene fragments were: from standard 1 (1:1 molar ratio of *Pfmdr1* and β-tubulin) to standard 4 (4:1 molar ratio of *Pfmdr-1* and β-tubulin). The 3D7 Africa line (which has one copy of *Pfmdr1*) and the Dd2line (which has three copies of *Pfmdr1*) were included in each run as controls.
Pfmdr1 copy number was calculated by the $2^{-\Delta Ct}$ method ($\Delta C_t = C_{t\ Pfmdr1} - C_{t\ Pf\beta\text{-}tubulin}$ where C_t is the threshold cycle) and deduced from the standard curve. A *Pfmdr1* copy number >1.6 was defined as an amplification of the gene. Amplification efficiencies of the *Pfmdr1* and the *Pf*-

tubulin genes measured by using ten-fold dilutions of 3D7 DNA, were similar (95% and 95%, respectively).

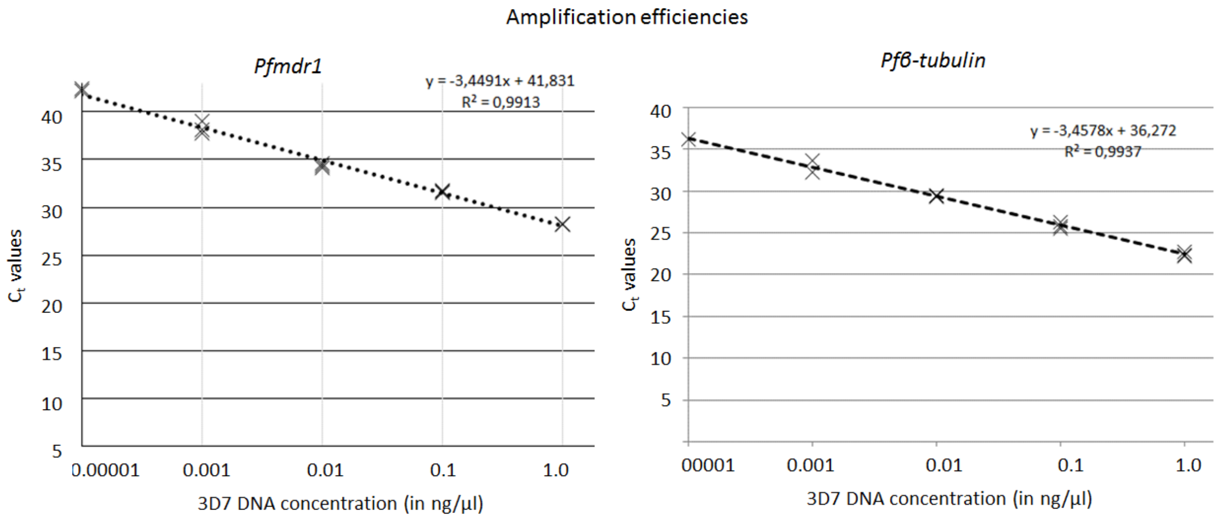

Appendix 3: *PfPM2* mRNA expression profile, with listing of primers, protocols, RT-qPCR efficiencies.

One step reverse transcriptase (RT)-qPCRs were carried out using a CFX96 real-time PCR machine (Bio-Rad) in 25 µl volumes in a 96-well plate containing 2X SuperScript™ III Platinum One step qRT-PCR kit (Life Technologies, Courtaboeuf, France), 0.2 µM concentrations of each forward and reverse primers, 0.1 µM concentrations of specific probes (FAM-BHQ1) and 3 µl of DNase-treated RNA.Listing of primers, protocols and PCR amplification efficiencies are provided in the table below.

	**Primer Sequence**	**Sequences**	**Tm (°C)**	**Product size (bp)**
RT-PCR *PfPM2*	PfPM2_RTPCR_F	5'-GGATTCGAACCAACTTATACTGC-3'	59.1	90
	PfPM2_RTPCR_R	5'-AATTGGATCTACTGAACCTATTGATAA-3'	57.9	
	PfPM2_RTPCR_Probe	5'-FAM-CAACATTTGATGGTATCCTTGGTTTAGGATGGA-BHQ1-3'	71.3	
	Pfserine-tRNA ligase_RTPCR_F	5'-TGGAACAATGGTAGCTGCAC-3'	59.7	92
	Pfserine-tRNA ligase_RTPCR_R	5'-GGCGCAATTTTTCAGGAACT-3'	61.5	
	Pfserine-tRNA ligase_RTPCR_Probe	5'-FAM-TGTCTTCTTGAAAATTATCAAAACGGCGAAGG-BHQ1-3'	71.6	

Amplifications were performed under the following conditions: 50°C for 15min, and 95°C for 2min, followed by 35 cycles of 95°C for 15s, 60°C for 30s and a final cycle at 35°C for 30s. Fluorescence data were collected during the 60°C annealing-extension steps.

For each run, *PfPM2* and *Pfserine-tRNA ligase* mRNAs expression were measured in triplicate for each sample. DNase-treated RNA from 3D7 parasites (collected at trophozoite stage, 24h post-invasion) was included in each run as control. *PfPM2* mRNA expression, normalized to *Pfserine-tRNA ligase* mRNA expression, was calculated by the $2^{-\Delta\Delta Ct}$ method, using the following formula: $\Delta\Delta C_t = [(C_{t\ PfPM2} - C_{t\ Pfserine\text{-}tRNA\ ligase})_{sample} - (C_{t\ PfPM2} - C_{t\ Pfserine\text{-}tRNA\ ligase})_{3D7}]$, where C_t is the threshold cycle.

RT-qPCR amplification efficiencies of *Pfplasmepsin2* and *Pfserine-tRNA ligase,* measured using ten-fold dilutions of DNase-treated 3D7 RNA, were similar (104% and 107%, respectively).

RT-qPCR efficiencies

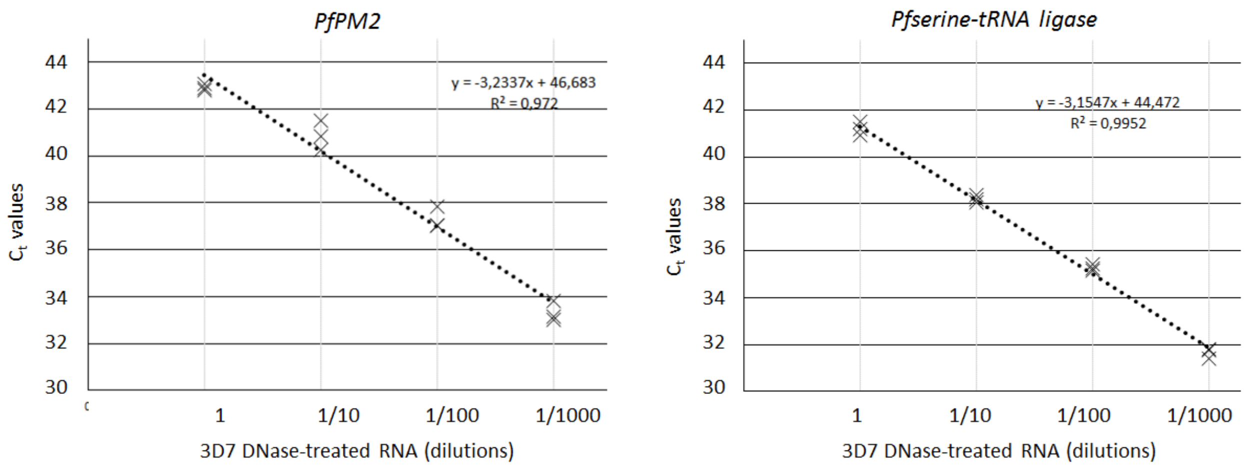

Appendix 4:

Panel A. Manhattan plot showing the significance of single nucleotide polymorphisms (SNPs) between whole-genome exome sequences of 23 piperaquine-resistant and 8 piperaquine-sensitive culture-adapted lines phenotyped using in-vitro PSA.

Each dot represents a SNP in a set of 31 culture-adapted parasites, according to chromosome. The x axis represents genomic location, and the y axis represents the log_{10} transformed Fischer exact test's p-values. After Bonferroni correction at level 5%, only 2 positions in 2 genes, position 896588 of PF3D7_0420000 (zinc finger protein, putative) and position 908385 of PF3D7_0420100 (serine/threonine protein kinase RIO2) achieved genome-wide significance between the resistant and sensitive lines ($p<3.56x10^{-7}$ for both SNP, Fisher's exact test) [$>6.38=log_{10}$ (120691/0.05)] (see Panel B for details).

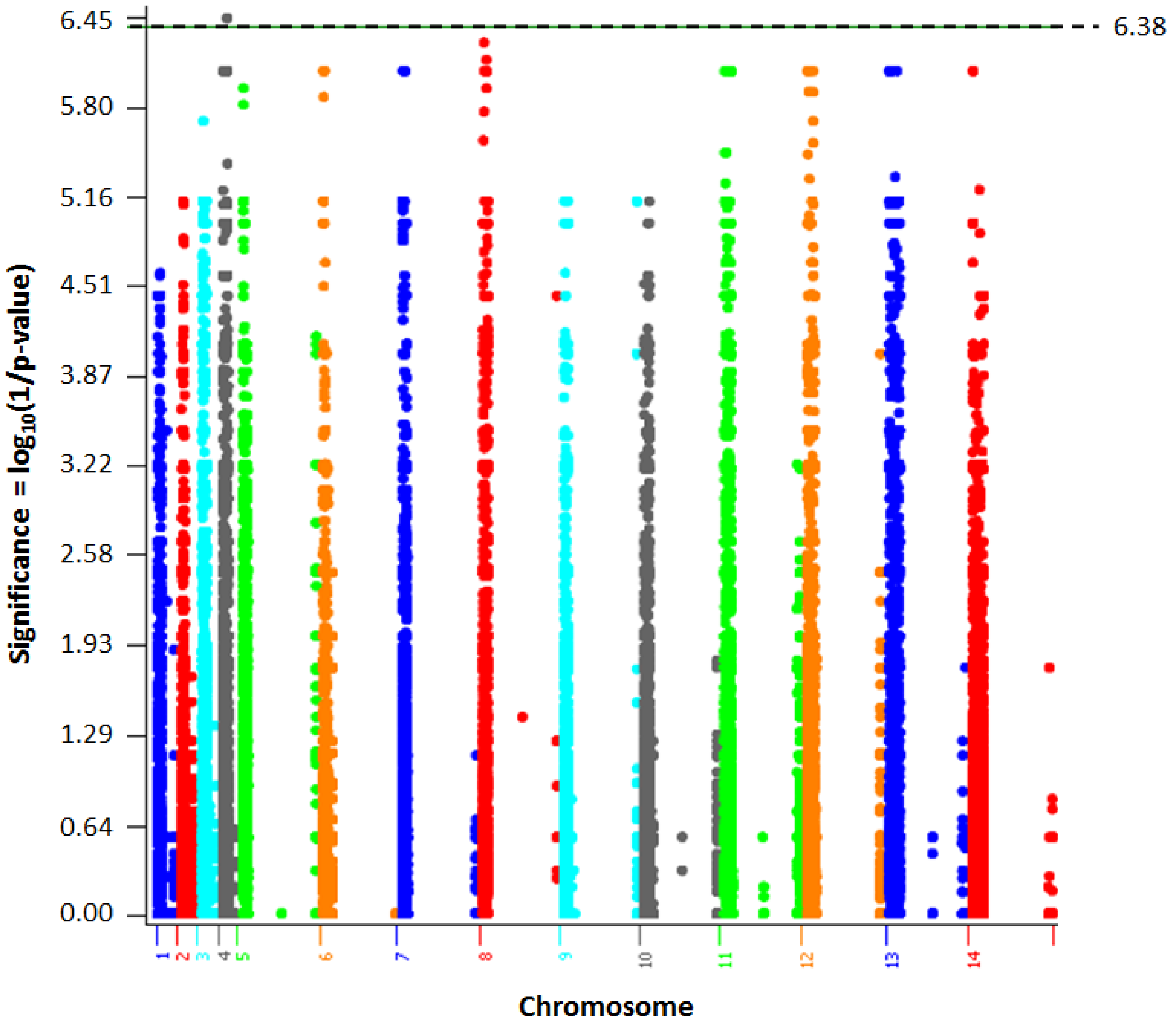

Panel B. List of the positions with variable proportions of wild type and mutant nucleotides of PF3D7_0420000 (encoding a putative zinc finger protein) and PF3D7_0420100 (encoding the Rio2 serine/threonine protein kinase) sequences of 23 piperaquine-resistant (red background) and 8 piperaquine-sensitive (green background) culture-adapted lines phenotyped using in-vitro PSA (Multalin online software, http://multalin.toulouse.inra.fr/multalin/).

SNP (position 896588) in PF3D7_0420000 gene (encoding a putative zinc finger protein) ($p<3.56 \times 10^{-7}$, Fisher's exact test) and SNP (position 908385) in PF3D7_0420100 gene (encoding the Rio2 serine/threonine protein kinase) ($p<3.56 \times 10^{-7}$, Fisher's exact test) detected as significant between piperaquine-resistant and piperaquine-sensitive parasite lines are shown in red bold font

Nucleotide code (IUPAC nomenclature): A: Adenine, C: Cytosine, G: Guanine, T: Thymine, R: A or G, Y: C or T, S: G or C, W: A or T, K: G or T, M: A or C, B: C or G or T, D: A or G or T, H: A or C or T, V: A or C or G, N: any base. If an indel is observed (>20% of the genome-wide mean coverage), letters are shown in lowercase.

Analysis ware done as follows:

For each position with a coverage >100, the variants with >20% frequency were included in the analysis (A, C, G, T or short INDEL). This results in a total of 30 possible values: 15 IUPAC code with upper case when no INDEL was detected and 15 with lower case when an INDEL was detected. For example, if a given position contains 17% A, 27% C, 32% G, 2% T, and 21% of reads with INDEL, the IUPAC code for this position and for this particular sample is « s ».

In particular, at position 896 588 of the gene PF3D7_0420000 (first table, line highlighted in red font), we observed 2 C and 7 Y in the sensitive lines (the 3D7 reference sequence was included because its sequence information is accurate for SNPs) and 3 C and 20 y in the resistant lines. Thus the contingency table and the Fisher exact probability test (3x2) results were:

	C	Y	y
Sensitive	2	7	0
Resistant	3	0	20

We found a significant p value of $3.56.\ 10^{-7}$, (p=0.042 after Bonferroni correction, for 120691 tests).

At position 908 385 of the gene PF3D7_0421000 (second table, line highlighted in red font), we observed 8 A and 1 m in the sensitive lines and 1 A and 22 a in the resistant lines. Thus the contingency table and the Fisher exact probability test (3x2) results were:

	A	m	a
Sensitive	8	1	0
Resistant	1	0	22

We also found a significant p value of $3.56.\ 10^{-7}$ (p=0.042 after Bonferroni correction, for 120691 tests).

PF3D7_0420000 (encoding a putative zinc finger protein)

Position on chromosome 4	Parasite line ID																															
	3D7	6273	6337	6267	6403	6349	6237	6410	6369	6395	6341	6280	6246	6293	6391	6272	6218	6302	6229	6443	6430	6365	6429	6394	6219	6408	6224	6431	6320	6261	6411	6427
	in-vitro PSA survival rate (%)																															
	0.1	0.2	0.4	0.5	0.5	0.6	0.8	6.0	6.4	19.2	25.8	28.9	36.9	39.3	39.4	40.0	40.8	42.5	46.6	49.6	51.3	51.8	51.8	56.7	58.6	58.7	61.4	61.5	62.1	70.5	71.6	77.4
	Mean Coverage (x)																															
	435	242	200	134	278	231	202	243	132	257	369	200	125	194	243	237	198	139	188	166	210	223	179	273	224	58	263	157	138	294	199	216
895028	A	A	A	M	A	A	A	A	A	A	A	A	A	A	A	A	A	A	A	A	A	A	A	A	A	A	A	A	A	A	A	A
895040	C	M	M	M	M	M	M	C	M	C	C	M	M	M	M	M	C	M	M	M	M	M	M	M	M	M	M	M	M	M	M	M
895055	G	R	R	R	R	R	R	G	R	G	G	R	R	R	R	R	G	R	R	R	R	R	R	R	R	R	R	R	R	R	R	R
895063	A	W	W	W	W	W	W	A	W	A	A	W	W	W	W	W	A	W	W	W	W	W	W	W	W	W	W	W	W	W	W	W
895067	A	R	R	R	R	R	R	A	R	A	A	R	R	R	R	R	A	R	R	R	R	R	R	R	R	R	R	R	R	R	R	R
895099	A	W	W	W	W	W	W	A	W	A	A	W	W	W	W	W	A	W	W	W	W	W	W	W	W	W	W	W	W	W	W	W
895111	T	W	W	W	W	W	W	T	W	T	T	W	W	W	W	W	T	W	W	W	W	W	W	W	W	W	W	W	W	W	W	W
895131	G	G	G	G	G	G	G	G	G	G	G	A	G	A	A	A	G	A	A	A	A	A	A	A	A	A	A	A	A	A	A	A
895785	A	A	A	A	A	A	A	A	A	A	A	A	A	A	A	A	A	A	A	A	A	A	A	A	A	W	A	A	A	A	A	A
896254	T	C	C	C	C	C	C	C	C	C	C	C	C	C	C	C	C	C	C	C	C	C	C	C	C	C	C	C	C	C	C	C
896321	C	A	A	A	A	A	A	C	A	C	C	C	C	C	C	C	C	C	C	C	C	C	C	C	C	C	C	C	C	C	C	C
896552	C	C	C	C	C	C	C	C	C	C	C	C	C	C	C	Y	C	C	C	C	C	C	C	C	C	C	Y	Y	C	Y	C	Y
896558	T	T	T	T	T	T	T	T	T	T	T	T	T	T	T	T	T	T	T	T	T	T	T	T	T	T	T	T	T	T	T	Y
896567	T	Y	Y	Y	Y	Y	Y	T	Y	T	T	T	T	T	T	T	T	T	T	T	T	T	T	T	T	T	T	T	T	T	T	T
896573	C	Y	Y	Y	Y	Y	Y	C	Y	C	C	C	Y	C	C	C	C	C	C	C	C	C	C	C	C	C	C	C	C	C	C	C
896585	T	T	T	T	T	T	T	T	T	T	T	t	T	t	t	t	T	T	T	t	t	t	t	t	t	t	t	t	t	t	t	t
896586	A	A	A	A	A	A	A	A	A	A	A	a	A	a	a	a	A	A	A	a	a	a	a	a	a	a	a	a	a	a	a	a
896587	T	T	T	T	T	T	T	T	T	T	T	t	T	t	t	t	T	T	T	t	t	t	t	t	t	t	t	t	t	t	t	t
896588	C	Y	Y	Y	Y	Y	Y	C	Y	C	C	y	y	y	y	y	C	y	y	y	y	y	y	y	y	y	y	y	y	y	y	y
896804	C	C	C	C	C	C	C	C	C	C	C	T	T	T	T	T	C	T	T	T	T	T	T	T	T	T	T	T	T	T	T	T
897069	A	A	A	A	A	A	A	W	A	A	A	A	A	A	W	A	A	A	A	A	A	A	A	A	A	A	A	A	A	A	A	A
897087	A	A	A	A	W	W	A	A	A	A	W	W	A	W	W	A	A	A	A	A	A	A	A	A	A	A	W	A	A	W	A	W
897094	A	W	W	W	A	W	A	A	W	W	W	W	W	W	W	W	A	W	A	A	A	W	A	A	A	A	W	W	A	W	A	A
897095	G	G	K	G	G	K	G	K	G	G	K	G	G	G	G	G	G	G	G	G	G	G	G	G	G	G	G	G	G	G	G	G
897097	A	W	A	A	A	A	A	A	A	A	A	A	A	W	A	A	A	A	A	A	A	A	A	A	A	A	A	A	A	A	A	A
897114	G	G	G	G	G	G	G	G	G	G	G	G	G	G	G	G	G	G	G	G	G	G	G	G	G	K	G	G	G	G	G	G
897121	G	G	G	G	G	G	G	G	G	G	G	G	G	G	G	G	G	G	G	G	G	G	G	G	G	K	G	G	G	G	G	G
897165	A	G	G	G	G	G	G	A	G	A	A	A	A	A	A	A	A	A	A	A	A	A	A	A	A	A	A	A	A	A	A	A
897266	C	G	G	G	G	G	G	C	G	C	C	C	C	C	C	C	C	C	C	C	C	C	C	C	C	C	C	C	C	C	C	C
897268	A	T	T	T	T	T	T	A	T	A	A	A	A	A	A	A	A	A	A	A	A	A	A	A	A	A	A	A	A	A	A	A
897275	T	T	T	T	T	T	T	T	T	T	T	T	T	T	T	T	T	Y	T	T	T	T	T	T	T	T	T	T	T	T	T	T
897284	C	G	G	G	G	G	G	C	G	C	C	C	C	C	C	C	C	C	C	C	C	C	C	C	C	C	C	C	C	C	C	C
897286	A	T	T	T	T	T	T	A	T	A	A	A	A	A	A	A	A	A	A	A	A	A	A	A	A	A	A	A	A	A	A	A
897293	T	Y			C	Y		T		T	T	T	T	T	T	T	T	T	T	T	T	T	T	T	T	T	T	T	T	T	T	T

Position on chromosome 4	Parasite line ID																															
	3D7	6273	6337	6267	6403	6349	6237	6410	6369	6395	6341	6280	6246	6293	6391	6272	6218	6302	6229	6443	6430	6365	6429	6394	6219	6408	6224	6431	6320	6261	6411	6427
	in-vitro PSA survival rate (%)																															
	0.1	0.2	0.4	0.5	0.5	0.6	0.8	6.0	6.4	19.2	25.8	28.9	36.9	39.3	39.4	40.0	40.8	42.5	46.6	49.6	51.3	51.8	51.8	56.7	58.6	58.7	61.4	61.5	62.1	70.5	71.6	77.4
	Mean Coverage (x)																															
	435	242	200	134	278	231	202	243	132	257	369	200	125	194	243	237	198	139	188	166	210	223	179	273	224	58	263	157	138	294	199	216
897296	C	C			T	T		C		C	C	C	C	C	C	C	C	C	C	C	C	C	C	C	C	C	C	C	C	C	C	C
897297	C	C						C		C	C	C	C	C	C	C	C	C	C	C	C	C	C	C	C	C	C	C	C	C	C	C
897302	C	C						C		C	C	C	C	C	C	C	C	C	C	C	C	C	C	C	C	C	C	C	C	C	C	C
897304	A	W						W		W	W	W	W	W	T	W	W	W	W	W	W	W	T	W	W	W	T	W	W	W	W	T
897305	C	C						C		C	C	C	C	C	C	C	C	C	C	C	C	C	C	C	C	C	C	C	C	C	C	C
897306	C	C						C		C	C	C	C	C	C	C	C	C	C	C	C	C	C	C	C	C	C	C	C	C	C	C
897314	C	Y	C			T		C		C	C	C	C	C	C	C	C	C	C	C	C	C	C	C	C	C	C	C	C	C	C	C
897315	C	Y	C			T		C		C	C	C	C	C	C	C	C	C	C	C	C	C	C	C	C	C	C	C	C	C	C	C
897320	C	C	C	C	C	C	C	Y	C	Y	Y	Y	Y	Y	Y	Y	Y	Y	Y	Y	Y	Y	Y	Y	Y	Y	Y	Y	Y	Y	Y	Y
897329	T	G	G	G	G	G	G	K	G	K	K	K	K	K	K	K	K	K	K	K	K	K	K	K	K	K	K	K	K	K	K	K
897338	C	T	T	T	T	T	T	Y	T	Y	Y	Y	Y	Y	Y	Y	Y	Y	Y	Y	Y	Y	Y	Y	Y	Y	Y	Y	Y	Y	Y	Y
897347	C	G	K	G	G	G	G	K	K	K	K	G	K	K	K	G	G	G	G	G	G	G	K	K	G	G	G	K	G	K	G	K
897358	C	T	T	T	T	T	T	T	T	T	T	T	T	T	T	T	T	T	T	T	T	T	T	T	T	T	T	T	T	T	T	T
897360	G	G	G	G	G	G	G	G	G	G	G	G	K	G	G	G	G	G	G	G	G	G	K	G	G	G	G	G	G	G	G	G
897363	A	A	A	A	A	A	A	A	A	W	W	A	W	A	A	A	A	A	A	A	A	A	W	A	A	A	A	A	A	A	A	W
897371	A	A	A	A	A	A	A	A	A	A	A	A	W	A	A	A	A	A	A	A	A	A	A	A	A	A	A	A	A	A	A	A
897378	A	A	A	A	A	A	A	A	A	A	A	A	W	A	A	W	A	A	A	A	A	A	A	A	A	W	A	A	A	A	A	A
897390	G	G	G	G	G	G	G	G	G	G	G	G	G	G	K	G	G	G	G	G	K	G	G	G	G	G	G	K	G	G	G	G
897393	A	A	A	A	A	A	A	A	A	A	W	W	W	W	W	W	W	W	W	A	W	W	W	W	W	W	A	W	W	W	A	W
897419	T	C	C	C	C	C	C	T	C	T	T	T	T	T	T	T	T	T	T	T	T	T	T	T	T	T	T	T	T	T	T	T
897462	C	A	A	A	A	A	A	C	A	C	C	C	C	C	C	C	C	C	C	C	C	C	C	C	C	C	C	C	C	C	C	C
897468	C							C		C	C	C	C	C	C	C	C	C	C	C	C	C	C	C	C	C	C	C	C	C	C	C
898387	G	G	G	G	G	G	G	A	G	A	A	G	G	G	G	G	A	G	G	G	G	G	G	G	G	G	G	G	G	G	G	G
898407	A	C	C	C	C	C	C	C	C	C	C	C	C	C	C	C	C	C	C	C	C	C	C	C	C	C	C	C	C	C	C	C
898504	C	c	c	c	c	c	c	c	c	c	c	c	c	c	c	c	c	c	c	c	c	c	c	c	c	c	c	c	c	c	c	c
898522	C	Y	Y	Y	Y	Y	Y	Y	Y	Y	Y	Y	Y	Y	Y	Y	Y	Y	Y	Y	Y	Y	Y	Y	Y	Y	Y	Y	Y	Y	Y	Y
898528	T	Y	T	Y	Y	Y	Y	T	Y	T	T	T	T	T	T	T	T	T	T	T	T	T	T	T	T	T	T	Y	Y	T	T	T
898537	C	C	C	C	C	C	C	C	C	C	C	C	C	C	C	C	C	C	C	C	C	C	C	C	C	C	C	C	C	Y	Y	C
898538	C	C	C	C	C	C	C	C	C	C	C	C	Y	C	C	Y	C	Y	C	C	C	C	C	C	C	C	Y	C	C	Y	Y	C
898540	T	T	T	T	T	T	T	T	T	T	T	Y	Y	Y	Y	Y	T	Y	T	T	T	T	T	T	Y	Y	Y	T	Y	Y	Y	Y
898543	A	W	W	W	W	W	W	A	W	A	A	W	W	W	W	W	A	W	W	W	W	W	W	A	W	W	W	W	W	W	W	W
898555	C	c	c	c	c	c	c	C	c	C	C	C	C	C	C	C	C	C	C	C	C	C	C	C	C	C	C	C	C	C	C	C
898556	C	c	c	c	c	c	c	C	c	C	C	C	C	C	C	C	C	C	C	C	C	C	C	C	C	C	C	C	C	C	C	C
898557	A	a	a	a	a	a	a	A	a	A	A	A	A	A	A	A	A	A	A	A	A	A	A	A	A	A	A	A	A	A	A	A
898558	T	T	t	y	t	y	t	T	t	T	T	T	T	T	T	T	T	T	T	T	T	T	T	T	T	T	T	T	T	T	T	T
898561	A	W	W	W	W	W	W	A	W	A	A	A	A	A	A	A	A	A	A	A	A	A	A	A	A	A	A	A	A	A	A	A
898976	T	C	C	C	C	C	C	C	C	C	C	C	C	C	C	C	C	C	C	C	C	C	C	C	C	C	C	C	C	C	C	C
899306	T	T	T	T	T	T	T	T	T	T	T	T	Y	T	T	T	T	T	T	T	T	T	T	T	T	T	T	T	T	T	T	T
899339	G	G	G	G	C	G	G	G	C	G	G	G	G	G	G	G	G	G	G	G	G	G	G	G	G	G	G	G	G	G	G	G
899359	C	T	T	T	T	T	T	T	T	T	T	T	T	T	T	T	T	T	T	T	T	T	T	T	T	T	T	T	T	T	T	T

Position on chromosome 4	Parasite line ID																																				
	3D7	6273	6337	6267	6403	6349	6237	6410	6369	6395	6341	6280	6246	6293	6391	6272	6218	6302	6229	6443	6430	6365	6429	6394	6219	6408	6224	6431	6320	6261	6411	6427
	in-vitro PSA survival rate (%)																																				
	0.1	0.2	0.4	0.5	0.5	0.6	0.8	6.0	6.4	19.2	25.8	28.9	36.9	39.3	39.4	40.0	40.8	42.5	46.6	49.6	51.3	51.8	51.8	56.7	58.6	58.7	61.4	61.5	62.1	70.5	71.6	77.4
	Mean Coverage (x)																																				
	435	242	200	134	278	231	202	243	132	257	369	200	125	194	243	237	198	139	188	166	210	223	179	273	224	58	263	157	138	294	199	216
899379	A	T	T	T	T	T	T	A	T	A	A	T	T	T	T	T	A	T	T	T	T	T	T	T	T	T	T	T	T	T	T	T
899385	A	a	a	a	A	a	a	A	A	A	A	a	a	a	a	a	A	a	a	a	a	A	A	a	a	a	a	a	a	a	a	a
899386	T	Y	Y	Y		Y	Y	T	C	T	T	Y	Y	Y	Y	Y	T	Y	Y	Y	Y	C	C	Y	Y	T	Y	T	Y	T	Y	Y
899387	T	W	W	W		A	W	T	T	T	T	A	A	W	A	A	T	W	A	W	W	W	T	W	A	A	W	A	W	W	W	W
899388	T	M	M	M		C	M	T		T	T	C	C	M	C	C	T	H	C	M	M	M	M	M	C	C	M	C	C	Y	M	M
899389	T	Y	Y	Y		T	Y	T		T	T	T	T	T	Y	T	T	Y	T	Y	Y	C	Y	Y	T	T	Y	T	T	T	Y	Y
899390	G	T	Y	Y		Y	C	G		G	G	C	Y	C	C	C	G	K	C	T	C	T	T	Y	Y	C	Y	C	C	S	Y	Y
899391	A	A	A	A		A	A	A		A	A	A	A	A	A	A	A	A	A	A	A	A	A	A	A	A	A	A	A	A	A	A
899392	T	T	T	T		T	T	T		T	T	T	T	T	T	T	T	T	T	T	T	T	T	T	T	T	T	T	T	T	T	T
899393	C							C		C	C						C	C												C		
899394	A							A		A	A						A	A												A		
899399	G				C			G	C	G	G						G	G												G		
899400	A	A	A	A	A	A	A	A	A	A	A	A	A	A	A	A	A	A	A	A	A	A		A			A	A	A	A	A	A
899401	T	T	T	T	T	T	T	T	T	T	T	T	T	T	T	T	T	T	T	W	T	T	T	T	T		T	T	T	T	T	T
899402	C	G	G	G	C	G	G	C	C	C	C	G	G	G	G	G	C	G	G	S	G	G	G	G	G		G	G	G	S	G	G
899403	A	A	A	A	A	A	A	A	A	A	A	A	A	A	A	A	A	A	A	A	A	A	A	A	A		A	A	A	A	A	A
899404	C	T	T	T	T	T	T	Y	T	Y	Y	T	T	T	T	T	Y	T	T	T	T	T	T	T	T		T	T	T	Y	T	T
899405	T	A	A	A	A	A	A	T	A	T	T	A	A	A	A	A	T	A	A	A	A	A	A	A	A	A	A	A	A	W	A	A
899406	A	T	T	T	T	T	T	A	T	A	A	T	T	T	T	T	A	T	T	T	T	T	T	T	T	T	T	T	T	T	T	T
899408	G	C	C	C	C	C	C	G	C	G	G	C	C	C	C	C	G	C	C	C	C	C	C	C	C	C	C	C	C	C	C	C
899411	C	A	A	A	A	A	A	C	A	C	C	A	A	A	A	A	C	A	A	A	A	A	A	A	A	A	A	A	A	A	A	A
899915	T	T	T	T	T	T	T	T	T	T	T	T	Y	T	T	T	T	T	T	T	T	T	T	T	T	T	T	T	T	T	T	T
899970	T	T	T	T	T	T	T	T	T	T	T	T	Y	T	T	T	T	T	T	T	T	T	T	T	T	T	T	T	T	T	T	T
900145	A	T	T	T	A	T	T	A	A	A	A	A	A	A	A	A	A	A	A	A	A	A	A	A	A	A	A	A	A	A	A	A
900189	G	G	G	G	T	G	G	T	T	T	T	G	G	G	G	G	T	G	G	G	G	G	G	G	G	G	G	G	G	G	G	G
900191	C	C	C	C	T	C	C	T	T	T	T	C	C	C	C	C	T	C	C	C	C	C	C	C	C	C	C	C	C	C	C	C
900206	T	T	T	T	C	T	T	T	C	T	T	T	T	T	T	T	T	T	T	T	T	T	T	T	T	T	T	T	T	T	T	T
900208	A	A	A	A	T	A	A	A	T	A	A	A	A	A	A	A	A	A	A	A	A	A	A	A	A	A	A	A	A	A	A	A
900210	G	G	G	G	A	G	G	A	A	A	A	G	G	G	G	G	A	G	G	G	G	G	G	G	G	G	G	G	G	G	G	G
900213	A	A	A	A	T	A	A	T	T	T	T	A	A	A	A	A	T	A	A	A	A	A	A	A	A	A	A	A	A	A	A	A
900214	T	T	T	T	A	T	T	A	A	A	A	T	T	T	T	T	A	T	T	T	T	T	T	T	T	T	T	T	T	T	T	T
900218	G	G	G	G	T	G	G	T	T	T	T	G	G	G	G	G	T	G	G	G	G	G	G	G	G	G	G	G	G	G	G	G
900225	C	C	C	C	C	C	C	T	C	T	T	C	C	C	C	C	T	C	C	C	C	C	C	C	C	C	C	C	C	C	C	C
900229	A	A	A	A	T	A	A	W	T	W	W	A	A	A	A	A	W	A	A	A	A	A	A	A	A	A	A	A	A	A	A	A
900231	G	G	G	G	A	G	G	R	A	R	R	G	G	G	G	G	R	G	G	G	G	G	G	G	G	G	G	G	G	G	G	G
900242	C	C	C	C	C	C	C	S	C	S	S	C	C	C	C	C	S	C	C	C	C	C	C	C	C	C	C	C	C	C	C	C
900246	A	A	A	A	C	A	A	C	C	C	C	A	A	A	A	A	C	A	A	A	A	A	A	A	A	A	A	A	A	A	A	A
900252	G	G	G	G	A	G	G	A	A	A	A	G	G	G	G	G	A	G	G	G	G	G	G	G	G	G	G	G	G	G	G	G
900254	C	C	C	C	T	C	C	T	T	T	T	C	Y	C	C	C	T	C	C	C	C	C	C	C	C	C	C	C	C	C	C	C
900255	A	A	A	A	T	A	A	T	T	T	T	A	A	A	A	A	T	A	A	A	A	A	A	A	A	A	A	A	A	A	A	A

Position on chromosome 4	Parasite line ID																															
	3D7	6273	6337	6267	6403	6349	6237	6410	6369	6395	6341	6280	6246	6293	6391	6272	6218	6302	6229	6443	6430	6365	6429	6394	6219	6408	6224	6431	6320	6261	6411	6427
	in-vitro PSA survival rate (%)																															
	0.1	0.2	0.4	0.5	0.5	0.6	0.8	6.0	6.4	19.2	25.8	28.9	36.9	39.3	39.4	40.0	40.8	42.5	46.6	49.6	51.3	51.8	51.8	56.7	58.6	58.7	61.4	61.5	62.1	70.5	71.6	77.4
	Mean Coverage (x)																															
	435	242	200	134	278	231	202	243	132	257	369	200	125	194	243	237	198	139	188	166	210	223	179	273	224	58	263	157	138	294	199	216
900256	T	T	T	T	A	T	T	A	A	A	A	T	T	T	T	T	A	T	T	T	T	T	T	T	T	T	T	T	T	T	T	T
900260	G	G	G	G	T	G	G	T	T	T	T	G	G	G	G	G	T	G	G	G	G	G	G	G	G	G	G	G	G	G	G	G
900267	A	A	A	A	A	A	A	A	A	A	A	A	M	A	A	A	A	A	A	A	A	A	A	A	A	A	A	A	A	A	A	A
900273	G	G	G	G	K	G	G	T	K	T	T	G	G	G	G	G	T	G	G	G	G	G	G	G	G	G	G	G	G	G	G	G
900275	C	C	C	C	T	C	C	T	T	T	T	C	Y	C	C	C	T	C	C	C	C	C	C	C	C	C	C	C	C	C	C	C
900281	G	G	G	G	G	G	G	G	G	G	G	G	K	G	G	G	G	G	G	G	G	G	G	G	G	G	G	G	G	G	G	G
900284	C	C	C	C	S	C	C	C	S	C	C	C	C	C	C	C	C	C	C	C	C	C	C	C	C	C	C	C	C	C	C	C
900290	T	T	T	T	Y	T	T	T	Y	T	T	T	T	T	T	T	T	T	T	T	T	T	T	T	T	T	T	T	T	T	T	T
900292	A	A	A	A	W	A	A	A	W	A	A	A	A	A	A	A	A	A	A	A	A	A	A	A	A	A	A	A	A	A	A	A
900298	A	A	A	A	A	A	A	A	A	A	A	A	W	A	A	A	A	A	A	A	A	A	A	A	A	A	A	A	A	A	A	A
900309	T	T	T	T	M	T	T	T	M	T	T	T	T	T	T	T	T	T	T	T	T	T	T	T	T	T	T	T	T	T	T	T
900313	T	T	T	T	W	T	T	T	W	T	T	T	T	T	T	T	T	T	T	T	T	T	T	T	T	T	T	T	T	T	T	T
900315	A	A	A	A	W	A	A	A	W	A	A	A	A	A	A	A	A	A	A	A	A	A	A	A	A	A	A	A	A	A	A	A
900318	A	A	A	A	A	A	A	A	A	A	A	W	A	A	A	A	A	A	A	A	A	A	A	A	A	A	A	A	A	A	A	A
900323	G	G	G	K	g	K	G	G	g	G	G	K	G	K	G	G	G	G	G	G	G	G	G	K	G	G	G	G	G	G	K	G
900324	T	T	T	T	t	T	T	T	t	T	T	T	T	T	T	T	T	T	T	T	T	T	T	T	T	T	T	T	T	T	T	T
900325	T	T	T	T	t	T	T	T	t	T	T	T	T	T	T	T	T	T	T	T	T	T	T	T	T	T	T	T	T	T	T	T
900326	C	C	C	C	c	C	C	C	c	C	C	C	C	C	C	C	C	C	C	C	C	C	C	C	C	C	C	C	C	C	C	C
900327	G	G	G	G	g	G	G	G	g	G	G	G	G	G	G	G	G	G	G	G	G	G	G	G	G	G	G	G	G	G	G	G
900328	T	T	T	T	t	T	T	T	t	T	T	T	T	T	T	T	T	T	T	T	T	T	T	T	T	T	T	T	T	T	T	T
900329	C	C	C	C	c	C	C	C	c	C	C	C	C	C	C	C	C	C	C	C	C	C	C	C	C	C	C	C	C	C	C	C
900330	C	C	C	C	c	C	C	C	c	C	C	C	C	C	C	C	C	C	C	C	C	C	C	C	C	C	C	C	C	C	C	C
900331	A	A	A	A	a	A	A	A	a	A	A	A	A	A	A	A	A	A	A	A	A	A	A	A	A	A	A	A	A	A	A	A
900332	c	C	C	C	c	C	C	C	c	C	C	C	C	C	C	C	C	C	C	C	C	C	C	C	C	C	C	C	C	C	C	C
900333	T	T	T	T	a	T	T	A	a	A	A	T	T	T	T	T	A	T	T	T	T	T	T	T	T	T	T	T	T	T	T	T
900334	T	T	T	T	t	T	T	T	t	T	T	T	T	T	T	T	T	T	T	T	T	T	T	T	T	T	T	T	T	T	T	T
900335	T	T	T	T	t	T	T	T	t	T	T	T	T	T	T	T	T	T	T	T	T	T	T	T	T	T	T	T	T	T	T	T
900336	T	T	T	T	w	T	T	W	w	W	W	T	T	T	T	T	W	T	T	T	T	T	T	T	T	T	T	T	T	T	T	T
900337	T	T	T	T	t	T	T	T	t	T	T	T	T	T	T	T	T	T	T	T	T	T	T	T	T	T	T	T	T	T	T	T
900338	T	T	T	T	t	T	T	T	t	T	T	T	T	T	T	T	T	T	T	T	T	T	T	T	T	T	T	T	T	T	T	T
900339	T	T	T	T	w	T	T	W	w	W	W	T	T	T	T	T	W	T	T	T	T	T	T	T	T	T	T	T	T	T	T	T
900340	T	T	T	T	w	T	T	W	w	W	W	T	T	T	T	T	W	T	T	T	T	T	T	T	T	T	T	T	T	T	T	T
900341	T	T	T	T	t	T	T	T	t	T	T	T	T	T	T	T	T	T	T	T	T	T	T	T	T	T	T	T	T	T	T	T
900342	T	T	T	T	t	T	T	T	t	T	T	T	T	T	T	T	T	T	T	T	T	T	T	T	T	T	T	T	T	T	T	T
900343	T	T	T	T	t	T	T	T	t	T	T	T	T	T	T	T	T	T	T	T	T	T	T	T	T	T	T	T	T	T	T	T
900344	T	T	T	T	k	T	T	K	k	K	K	T	T	T	T	T	K	T	T	T	T	T	T	T	T	T	T	T	T	T	T	T
900348	G	G	G	K	G	G	G	G	G	G	G	G	G	G	G	G	G	G	K	G	G	G	G	G	G	G	G	G	G	G	G	G
900353	C	T	T	T	C	T	T	T	C	T	T	T	T	T	T	T	T	T	T	T	T	T	T	T	T	T	T	T	T	T	T	T
900355	T	W	W	W	T	W	W	W	T	W	W	W	W	W	W	W	W	W	W	W	W	W	W	W	W	W	W	W	W	W	W	W
900357	A	W	A	A	A	A	A	A	A	A	A	A	W	A	W	A	A	A	A	A	A	W	W	A	A	A	W	W	A	W	A	W

Position on chromosome 4	Parasite line ID																															
	3D7	6273	6337	6267	6403	6349	6237	6410	6369	6395	6341	6280	6246	6293	6391	6272	6218	6302	6229	6443	6430	6365	6429	6394	6219	6408	6224	6431	6320	6261	6411	6427
	in-vitro PSA survival rate (%)																															
	0.1	0.2	0.4	0.5	0.5	0.6	0.8	6.0	6.4	19.2	25.8	28.9	36.9	39.3	39.4	40.0	40.8	42.5	46.6	49.6	51.3	51.8	51.8	56.7	58.6	58.7	61.4	61.5	62.1	70.5	71.6	77.4
	Mean Coverage (x)																															
	435	242	200	134	278	231	202	243	132	257	369	200	125	194	243	237	198	139	188	166	210	223	179	273	224	58	263	157	138	294	199	216
900361	A	W	W	W	A	W	W	A	A	A	A	A	W	A	A	W	A	A	W	A	A	W	W	A	A	A	A	W	W	W	W	W
900363	T	T	T	T	T	T	T	T	T	T	T	T	T	T	T	T	W	T	T	T	T	T	T	T	T	T	T	T	T	T	T	T
900372	C	T	T	T	C	T	T	T	C	T	T	T	T	T	T	T	T	T	T	T	T	T	T	T	T	T	T	T	T	T	T	T
900381	A	A	A	A	A	A	A	A	A	A	A	A	A	A	A	A	A	A	A	A	A	A	A	A	A	A	A	W	A	A	A	A
900386	G	G	G	K	G	G	K	G	G	G	G	G	G	G	G	G	G	K	K	G	K	G	G	G	K	G	G	K	K	K	K	G
900389	C	C	C	C	C	C	C	C	C	C	C	C	C	C	C	C	C	C	C	C	C	C	C	C	C	C	C	C	C	C	Y	C
900390	G	G	G	G	G	G	K	G	G	G	G	G	G	G	G	G	G	G	G	G	G	G	G	G	G	G	G	G	G	G	G	G
900395	C	c	c	c	C	c	c	C	C	C	C	c	c	c	c	c	C	c	C	c	c	c	c	c	c	c	c	c	c	c	c	c
900396	A	t	t	t	A	t	t	A	A	A	A	t	T	T	t	t	A	T	T	t	t	t	t	t	T	t	t	t	t	t	t	t
900399	A	T	T	T	A	T	T	A	A	A	A	T	T	T	T	T	A	W	T	T	T	T	T	T	T	T	T	T	T	T	T	T
900403	A	T	T	T	A	T	T	A	A	A	A	T	T	T	T	T	A	T	T	T	T	T	T	T	T	T	T	T	T	T	T	T
900457	G	G	G	G	G	G	G	G	G	G	G	G	G	G	G	G	G	G	G	G	G	G	G	G	G	K	G	G	G	G	G	G
900665	A	A	A	A	G	A	A	A	G	A	A	G	R	G	G	G	A	G	G	G	G	G	G	G	G	G	G	G	G	G	G	G
900780	G	G	G	G	G	G	G	A	G	A	A	A	A	A	A	A	A	A	A	A	A	A	A	A	A	A	A	A	A	A	A	A
900957	T	C	C	C	C	C	C	C	C	C	C	C	C	C	C	C	C	C	C	C	C	C	C	C	C	C	C	C	C	C	C	C
900988	A	C	C	C	C	C	C	C	C	C	C	C	C	C	C	C	C	C	C	C	C	C	C	C	C	C	C	C	C	C	C	C
901096	G	G	G	G	G	G	G	A	G	A	A	G	G	G	G	G	A	G	G	G	G	G	G	G	G	G	G	G	G	G	G	G
901097	G	G	G	G	G	G	G	C	G	C	C	G	G	G	G	G	C	G	G	G	G	G	G	G	G	G	G	G	G	G	G	G
901197	A	A	A	A	A	A	A	G	A	G	G	A	A	A	A	A	G	A	A	A	A	A	A	A	A	A	A	A	A	A	A	A
901321	A	A	A	A	a	A	A	A	a	A	A	A	A	A	A	A	A	A	A	A	A	A	A	A	A	A	A	A	A	A	A	A
901322	T	T	T	T	t	T	T	T	t	T	T	T	T	T	T	T	T	T	T	T	T	T	T	T	T	T	T	T	T	T	T	T
901323	A	A	A	A	a	A	A	A	a	A	A	A	A	A	A	A	A	A	A	A	A	A	A	A	A	A	A	A	A	A	A	A
901324	T	T	T	T	t	T	T	T	t	T	T	T	T	T	T	T	T	T	T	T	T	T	T	T	T	T	T	T	T	T	T	T
901325	T	T	T	T	t	T	T	T	t	T	T	T	T	T	T	T	T	T	T	T	T	T	T	T	T	T	T	T	T	T	T	T
901326	A	A	A	A	a	A	A	A	a	A	A	A	A	A	A	A	A	A	A	A	A	A	A	A	A	A	A	A	A	A	A	A
901327	T	T	T	T	t	T	T	T	t	T	T	T	T	T	T	T	T	T	T	T	T	T	T	T	T	T	T	T	T	T	T	T
901328	T	T	T	T	t	T	T	T	t	T	T	T	T	T	T	T	T	T	T	T	T	T	T	T	T	T	T	T	T	T	T	T
901329	A	A	A	A	t	A	A	A	t	A	A	A	A	A	A	A	A	A	A	A	A	A	A	A	A	A	A	A	A	A	A	A
901330	T	T	T	T	a	T	T	T	a	T	T	T	T	T	T	T	T	T	T	T	T	T	T	T	T	T	T	T	T	T	T	T
901345	T	T	T	T	T	T	T	t	T	t	t	T	T	T	T	T	t	T	T	T	T	T	T	T	T	T	T	T	T	T	T	T
901408	A	T	T	T	T	T	T	T	T	T	T	T	T	T	T	T	T	T	T	T	T	T	T	T	T	T	T	T	T	T	T	T
901416	C	A	A	A	C	A	A	C	C	C	C	C	C	C	C	C	C	C	C	C	C	C	C	C	C	C	C	C	C	C	C	C
901429	T	T	T	T	C	T	T	T	C	T	T	C	C	C	C	C	T	C	C	C	C	C	C	C	C	C	C	C	C	C	C	C
901771	T	T	T	T	T	T	T	C	T	C	C	T	T	T	T	T	C	T	T	T	T	T	T	T	T	T	T	T	T	T	T	T
901913	G	A	A	A	A	A	A	A	A	A	A	A	A	A	A	A	A	A	A	A	A	A	A	A	A	A	A	A	A	A	A	A
902548	C	T	T	T	T	T	T	T	T	T	T	T	T	T	T	T	T	T	T	T	T	T	T	T	T	T	T	T	T	T	T	T
902591	G	C	C	C	C	C	C	G	C	G	G	C	C	C	C	C	G	C	C	C	C	C	C	C	C	C	C	C	C	C	C	C
902609	C	c	c	c	c	c	c	C	c	c	C	c	c	c	y	c	C	y	c	y	c	c	c	C	c	Y	c	c	c	c	c	y
902610	A	a	a	a	a	a	a	A	a	a	A	a	a	a	a	a	A	a	a	a	a	a	a	a	a	A	a	a	a	a	a	a
902611	T	t	t	t	t	t	t	T	t	t	T	t	t	t	t	t	T	t	t	t	t	t	t	t	t	T	t	t	t	t	t	t

Position on chromosome 4	Parasite line ID																															
	3D7	6273	6337	6267	6403	6349	6237	6410	6369	6395	6341	6280	6246	6293	6391	6272	6218	6302	6229	6443	6430	6365	6429	6394	6219	6408	6224	6431	6320	6261	6411	6427
	in-vitro PSA survival rate (%)																															
	0.1	0.2	0.4	0.5	0.5	0.6	0.8	6.0	6.4	19.2	25.8	28.9	36.9	39.3	39.4	40.0	40.8	42.5	46.6	49.6	51.3	51.8	51.8	56.7	58.6	58.7	61.4	61.5	62.1	70.5	71.6	77.4
	Mean Coverage (x)																															
	435	242	200	134	278	231	202	243	132	257	369	200	125	194	243	237	198	139	188	166	210	223	179	273	224	58	263	157	138	294	199	216
902612	T	t	t	t	t	t	t	T	t	t	T	t	t	t	y	y	T	y	t	y	y	t	y	T	t	Y	t	t	t	t	t	y
902613	A	A	A	A	A	A	A	A	A	a	A	A	A	A	A	A	A	A	A	A	A	A	A	A	A	A	A	A	A	A	A	A
902615	T	T	T	T	T	T	T	T	T	T	T	T	T	T	T	T	K	T	T	T	T	T	T	T	T	T	T	T	T	T	T	T
902624	T	Y	Y	Y	Y	Y	Y	T	T	T	T	Y	Y	Y	T	Y	T	Y	Y	Y	Y	Y	Y	Y	Y	Y	Y	Y	Y	Y	Y	Y
902627	T	Y	Y	Y	Y	Y	Y	T	Y	T	T	Y	Y	Y	Y	Y	T	Y	Y	Y	Y	Y	Y	Y	Y	Y	Y	Y	Y	Y	Y	Y
902633	T	T	T	T	T	T	T	Y	T	Y	Y	T	T	T	T	T	Y	T	T	T	T	T	T	T	T	T	T	T	T	T	T	T
902642	C	C	C	C	C	C	C	T	C	Y	T	C	C	Y	C	C	T	C	C	C	C	C	C	C	C	C	C	C	C	C	C	C
902663	C	T	T	T	T	T	T	T	T	Y	T	T	T	T	T	T	T	T	T	T	T	T	T	T	T	T	T	T	T	T	T	T
903129	A	A	A	A	A	A	A	A	A	A	A	A	A	A	A	A	A	A	A	A	A	A	A	A	W	A	A	A	A	A	A	A
903141	A	A	A	A	W	A	A	A	W	A	A	A	A	A	A	A	A	A	A	A	A	A	A	A	A	A	A	A	A	A	A	A
903155	A	A	A	A	W	A	A	A	A	A	A	A	A	A	A	A	A	A	A	W	A	A	A	A	W	A	A	A	A	A	A	A
903161	C	C	C	C	C	C	C	Y	C	C	Y	C	Y	C	C	C	C	C	Y	Y	Y	C	Y	C	C	Y	C	Y	C	Y	C	Y
903176	G	K	K	K	K	K	K	K	K	K	K	K	K	K	K	K	K	K	K	G	G	K	K	K	K	K	K	K	K	K	K	K
903181	A	A	A	A	A	A	A	A	A	A	A	A	A	A	A	A	A	W	A	A	A	A	A	A	A	A	A	A	A	A	A	A
903182	A	A	A	A	A	A	A	A	A	A	A	A	A	A	A	A	A	W	A	A	A	A	A	A	A	A	A	A	A	A	A	A
903183	A	A	A	A	A	A	A	A	A	A	A	A	A	A	A	A	A	W	A	A	A	A	A	A	A	A	A	A	A	A	A	A
903186	G	G	K	G	G	G	G	G	G	G	G	G	K	G	G	G	G	K	G	G	G	G	G	G	G	K	G	G	G	G	G	G
903187	G	G	G	G	G	G	G	G	G	G	G	G	G	G	G	G	G	K	G	G	G	G	G	G	G	G	G	G	G	G	G	G
903188	A	A	W	A	A	A	A	A	A	A	A	A	A	A	A	A	A	W	A	A	A	A	A	A	A	A	A	A	A	A	A	A
903207	G	A	A	A	A	A	A	A	A	A	A	A	A	A	A	A	A	A	A	A	A	A	A	A	A	A	A	A	A	A	A	A
903414	A	A	A	A	A	A	A	A	A	A	A	G	G	G	G	G	A	G	G	G	G	G	G	G	G	G	G	G	G	G	G	G
903891	G	G	G	G	G	G	G	T	G	T	T	G	G	G	G	G	T	G	G	G	G	G	G	G	G	G	G	G	G	G	G	G
903950	A	A	A	A	A	A	A	C	A	C	C	A	A	A	A	A	C	A	A	A	A	A	A	A	A	A	A	A	A	A	A	A
904088	G	G	G	G	G	G	G	G	G	G	G	A	A	A	A	A	G	A	A	A	A	A	A	A	A	A	A	A	A	A	A	A
904159	C	C	C	C	C	C	C	T	C	T	T	C	C	C	C	C	T	C	C	C	C	C	C	C	C	C	C	C	C	C	C	C
904629	G	G	G	G	G	K	G	G	G	K	G	G	K	G	G	G	G	G	G	G	G	G	G	G	G	G	G	K	G	G	G	G

PF3D7_0420100 (encoding the Rio2 serine/threonine protein kinase)

Position on chromosome 4	Parasite line ID																															
	3D7	6273	6337	6267	6403	6349	6237	6410	6369	6395	6341	6280	6246	6293	6391	6272	6218	6302	6229	6443	6430	6365	6429	6394	6219	6408	6224	6431	6320	6261	6411	6427
	in-vitro PSA survival rate (%)																															
	0.1	0.2	0.4	0.5	0.5	0.6	0.8	6.0	6.4	19.2	25.8	28.9	36.9	39.3	39.4	40.0	40.8	42.5	46.6	49.6	51.3	51.8	51.8	56.7	58.6	58.7	61.4	61.5	62.1	70.5	71.6	77.4
	Mean Coverage (x)																															
	272	232	214	132	325	242	203	198	131	203	237	121	143	132	179	169	127	88	125	117	109	167	146	152	150	31	209	109	67	250	104	172
907571	T	C	C	C	C	C	C	C	C	C	C	C	T	C	C	C	C	C	C	C	C	C	C	C	C	C	C	C	C	C	C	C
908347	T	A	A	A	A	A	A	A	A	A	A	T	A	T	T	T	A	T	T	T	T	T	T	T	T	T	T	T	T	T	T	T
908382	G	G	G	G	G	G	G	R	G	R	G	G	G	G	G	G	G	G	G	G	G	G	G	G	G	G	G	G	G	G	G	G
908385	A	A	A	A	A	A	A	m	A	a	a	a	A	a	a	a	a	a	a	a	a	a	a	a	a	a	a	a	a	a	a	a
908388	A	A	A	A	A	A	A	A	A	A	A	R	A	A	A	A	A	R	A	A	R	A	A	R	A	R	A	R	R	A	R	A
908391	C	C	C	C	C	C	C	C	C	C	C	M	C	C	C	C	M	M	C	C	M	C	C	M	C	M	C	M	M	C	M	C
908394	A	A	A	A	A	A	A	A	A	A	A	A	A	A	A	A	A	A	A	A	A	A	A	R	A	R	A	A	R	A	R	A
908458	G	g	g	g	g	g	g	G	g	G	G	G	g	G	G	G	G	G	G	G	G	G	G	G	G	G	G	G	G	G	G	G
908459	T	t	t	t	t	t	t	T	t	T	T	T	t	T	T	T	T	T	T	T	T	T	T	T	T	T	T	T	T	T	T	T
908460	G	a	a	a	a	a	a	G	a	G	G	g	a	g	g	g	G	g	g	g	g	g	g	g	g	g	g	g	g	g	g	g
908461	A	a	a	a	a	a	a	A	a	A	A	a	a	a	a	a	A	a	a	a	a	a	a	a	a	a	a	a	a	a	a	a
908462	T	T	T	T	T	T	T	T	T	T	T	t	T	t	t	t	T	t	t	t	t	t	t	t	t	t	t	t	t	t	t	t
908463	A	A	A	A	A	A	A	A	A	A	A	a	A	a	a	a	A	a	a	a	a	a	a	a	a	a	a	a	a	a	a	a
908464	A	A	A	A	A	A	A	A	A	A	A	a	A	a	a	a	A	a	a	a	a	a	a	a	a	a	a	a	a	a	a	a
908465	T	T	T	T	T	T	T	T	T	T	T	t	T	t	t	t	T	t	t	t	t	t	t	t	t	t	t	t	t	t	t	t
908466	A	A	A	A	A	A	A	A	A	A	A	a	A	a	a	a	A	a	a	a	a	a	a	a	a	a	a	a	a	a	a	a
908467	A	A	A	A	A	A	A	A	A	A	A	a	A	a	a	a	A	a	a	a	a	a	a	a	a	a	a	a	a	a	a	a
908468	T	T	T	T	T	T	T	T	T	T	T	t	T	t	t	t	T	t	t	t	t	t	t	t	t	t	t	t	t	t	t	t
908469	A	A	A	A	A	A	A	A	A	A	A	a	A	a	a	a	A	a	a	a	a	a	a	a	a	a	a	a	a	a	a	a
908470	A	A	A	A	A	A	A	A	A	A	A	a	A	a	a		A	a	a	a	a	a	a	a	a	a		a	g	a	a	a
908471	T	T	T	T	T	T	T	T	T	T	T	t	T	t	t	t	T	t	t	t	t	t	t	t	t	t	t	t	t	t	t	t
908472	A	A	A	A	A	A	A	A	A	A	A	a	A	a	a		A	a	a	a	a	a		a	a	g		a	g	a	a	
908685	A	G	G	G	G	G	G	A	G	A	A	G	G	G	G	G	A	G	G	G	G	G	G	G	G	G	G	G	G	G	G	G
908711	T	T	T	T	W	T	T	W	T	T	T	T	T	T	T	T	T	T	T	T	T	T	T	T	T	T	T	T	T	T	T	T
908713	G	G	G	G	G	G	G	G	G	G	G	G	G	G	G	R	G	G	G	G	G	G	G	G	G	G	G	G	G	G	G	G
908725	G	G	G	G	G	G	G	G	G	G	G	G	R	G	G	G	G	R	G	G	G	G	G	G	G	G	G	G	G	G	G	R
908726	T	T	T	T	T	T	T	W	T	W	W	T	W	T	W	T	T	T	T	T	W	T	W	T	T	T	W	W	T	W	T	W
908729	G	G	G	G	G	G	G	G	G	G	G	G	R	G	G	G	G	G	G	G	G	G	G	G	G	G	G	G	G	G	G	R
908730	G	G	G	G	G	G	G	R	G	R	G	G	R	G	G	G	G	G	R	G	G	G	R	G	G	G	G	G	G	G	G	G
908736	T	T	T	T	T	T	T	W	T	W	W	T	W	W	W	W	W	T	W	W	W	W	W	W	W	W	W	W	W	W	W	W
908738	T	T	T	T	W	T	T	W	W	W	W	T	W	W	W	W	W	W	W	W	W	W	W	W	W	W	W	T	W	W	W	W
908742	G	G	R	R	R	G	R	R	R	R	R	R	R	R	R	R	R	R	R	R	R	R	R	R	R	R	R	R	R	R	R	R
908747	T	T	T	W	W	T	T	T	W	W	W	W	W	W	W	W	T	T	T	W	T	T	W	W	W	W	W	T	W	W	W	W
908748	T	T	T	W	T	T	T	T	T	T	T	T	T	T	T	T	T	T	T	T	T	T	T	W	W	T	T	T	T	T	T	T

Position on chromosome 4	Parasite line ID																															
	3D7	6273	6337	6267	6403	6349	6237	6410	6369	6395	6341	6280	6246	6293	6391	6272	6218	6302	6229	6443	6430	6365	6429	6394	6219	6408	6224	6431	6320	6261	6411	6427
	in-vitro PSA survival rate (%)																															
	0.1	0.2	0.4	0.5	0.5	0.6	0.8	6.0	6.4	19.2	25.8	28.9	36.9	39.3	39.4	40.0	40.8	42.5	46.6	49.6	51.3	51.8	51.8	56.7	58.6	58.7	61.4	61.5	62.1	70.5	71.6	77.4
	Mean Coverage (x)																															
	272	232	214	132	325	242	203	198	131	203	237	121	143	132	179	169	127	88	125	117	109	167	146	152	150	31	209	109	67	250	104	172
908749	T	T	T	W	T	T	W	W	W	W	W	T	W	W	W	W	W	W	W	T	W	W	W	T	W	W	W	T	W	W	T	W
908755	C	M	M	M	M	M	M	M	M	M	M	M	M	M	M	M	M	M	M	M	M	M	M	M	M	M	M	M	M	M	M	M
908762	T	W	W	W	W	W	W	W	W	A	W	W	W	W	W	W	W	W	W	W	W	W	W	W	W	W	W	W	W	W	W	W
908772	G	r	R	R	R	R	G	R	G	R	R	R	G	R	R	R	G	R	G	R	G	R	R	G	R	R	R	R	R	R	G	R
908773	T	t	T	T	W	T	T	T	T	T	T	T	T	T	T	T	T	T	T	T	T	W	T	T	T	T	T	T	T	T	T	T
908774	T	t	T	T	W	T	T	T	T	T	T	T	T	T	T	T	T	W	T	T	T	T	T	T	T	T	T	T	T	T	T	T
908776	T	T	T	W	W	T	T	T	T	T	T	T	T	T	T	W	T	T	T	T	T	T	T	T	T	W	T	T	T	T	T	T
908778	G	G	R	G	R	G	G	G	G	G	G	G	G	G	G	G	G	G	G	G	G	G	G	G	G	G	G	G	G	G	G	G
908780	G	R	R	R	R	R	R	R	R	R	R	R	R	R	R	R	R	R	R	R	R	R	R	R	R	R	R	R	R	R	R	R
908785	T	W	W	W	W	T	T	W	T	W	W	W	W	W	W	W	W	W	W	W	W	W	W	W	W	W	W	W	W	W	W	W
908786	A	W	W	W	W	W	W	A	W	A	A	A	A	A	A	A	A	A	A	A	A	A	A	A	A	A	A	A	A	A	A	A
908792	T	T	W	T	W	W	W	W	T	T	T	W	T	T	T	W	T	T	T	T	T	T	T	T	T	W	T	T	T	W	T	T
908793	T	W	W	T	W	T	T	T	W	T	T	T	T	T	T	T	T	T	T	T	T	T	T	T	T	T	T	T	T	T	T	T
908794	A	A	A	M	A	A	A	A	A	A	A	A	A	A	A	A	A	A	A	A	A	A	A	A	A	A	A	A	A	A	A	A
908796	T	H	Y	H	H	M	y	T	Y	T	T	T	T	T	T	T	T	T	T	T	T	T	T	T	T	T	T	T	T	T	T	T
908797	T	W	W	T	W	Y	W	T	W	T	T	T	T	T	T	T	T	T	T	T	T	T	T	T	T	T	T	T	T	T	T	T
908799	T	W	W	W	W	T	W	W	W	W	W	W	W	T	W	W	W	T	W	T	W	W	W	W	W	W	W	W	W	W	T	W

Appendix 5: Profiles of DNA expansion in the region of genes encoding proteins involved in hemoglobin-degrading activities positively associated with in-vitro piperaquine resistance and methodology developed to confirm the four DNA expansion profiles.

We performed an unsupervised classification of the amplification profiles (50 genes flanking PfPM2-3) using a Gaussian mixture. Each class has its own prior proportion, a class-specific mean for each gene, and a global residual variance, which is shared across genes and classes (this unrealistic homoscedastic assumption was necessary to allow a good fitting of the mixture). Fitting was performed using the classical Expectation-Maximization algorithm (Dempster *et al.*, Journal of the Royal Statistical Society. Series B (Methodological), Vol. 39, No. 1. (1977), pp. 1-38) and in order to avoid sub-optimal solution we replicated the algorithm 50 times only retaining the best fitting. The mixture model was fitted for k=2, 3, 4, 5, 6 classes. Model selection was performed using the BIC (Bayesian Information Criterion). The selected model has k=3 classes, as shown below:

Model selection

	k=2	k=3	k=4	k=5	k=6
loglik	-250.102	-41.52158	132.9167	307.3057	430.4599
dim	103.000	157.0000	213.000	271.0000	331.0000
bic	1221.520	1182.524444	1225.8195	1283.2192	1457.0948

Specific signals of the three types of DNA expansion (types by parasite lines are given in Table 2)
(the x-axis represents the gene position, and the y-axis represents the amplification signal).

DNA expansion type 1

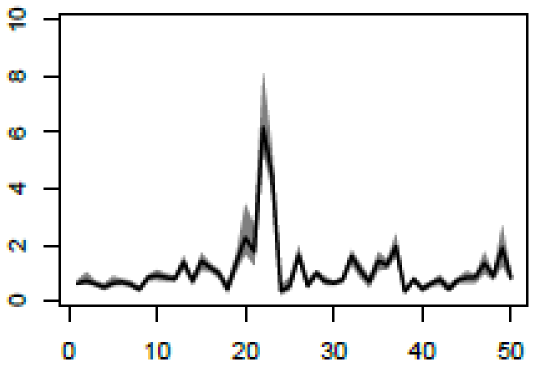

DNA expansion type 2

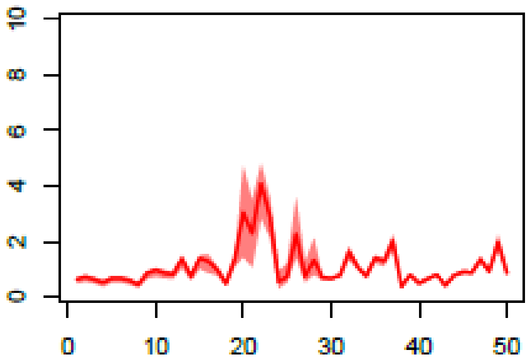

DNA expansion type 3

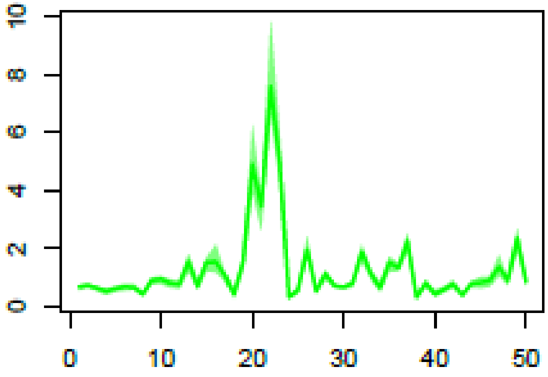

Appendix 6: Distribution of the Wilcoxon Rank-Sum test p-values ranking the significance of CNVs of the 4,616 genes screened between whole-genome exome sequences of 23 piperaquine-resistant and 8 piperaquine-sensitive culture-adapted lines phenotyped using in-vitro PSA.

Each dot represents a CNV. The x-axis represents the Wilcoxon rank-sum values, and the y-axis represents the p-values (Wilcoxon test). PF3D7_1408000 (*PfPM2*) and PF3D7_1408100 (*PfPM3*) ranked in the two first positions (p=2.43x10^{-5}). PF3D7_0523000 (*Pfmdr1*) was classified at the 4676th/4678 position (p=0.015).

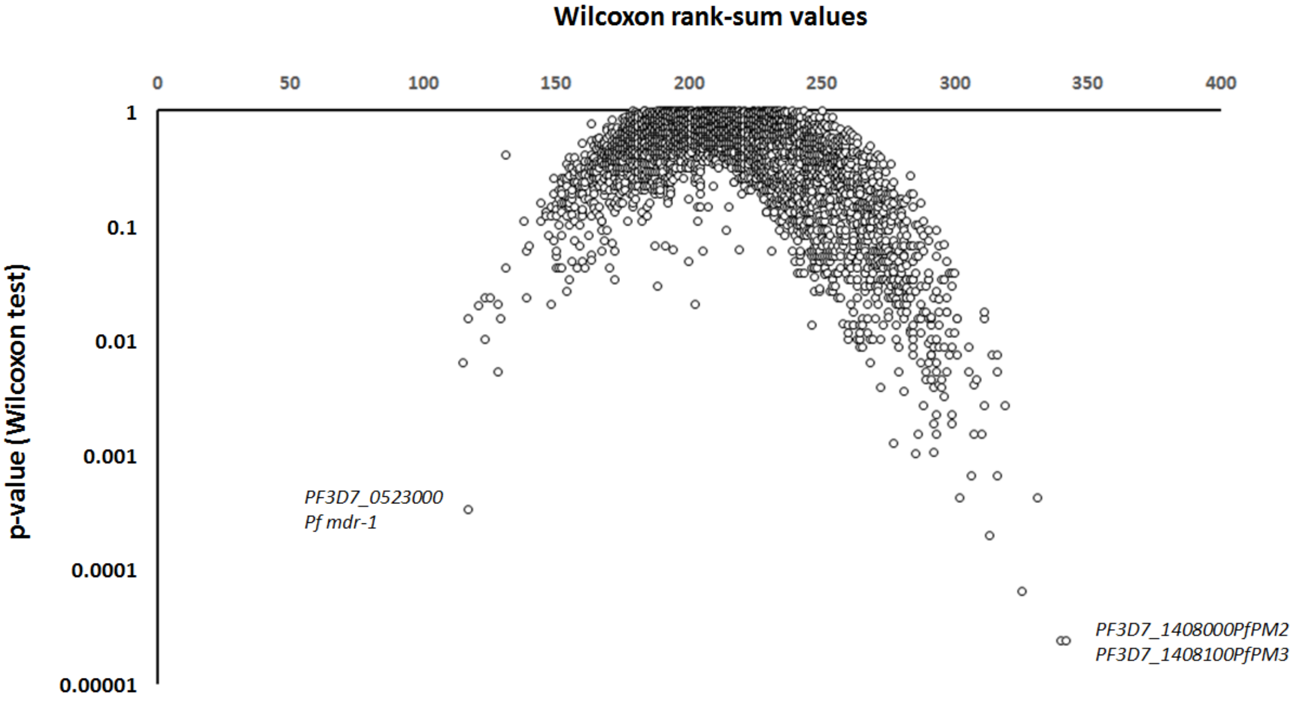

Appendix 7:

Panel A. *PfPM2* mRNA transcript levels, relative to *Pfserine-tRNA ligase* mRNA in early ring stages (H0: 0-3h post invasion), late ring stages (H12: 12-15h post invasion), early trophozoite stages (H24: 24-27h post invasion) and mature trophozoites (H36: 36-39h post invasion) of the in-vitro culture-adapted piperaquine-resistant ID_6320 line (PSA survival rate=62.1%, 2 copies *PfPM2,* C580Y K13 allele, solid red line) and piperaquine-sensitive ID_6267 line (PSA survival rate=0.5%, single copy *PfPM2,* C580Y K13 allele, solid black line) (see table 2 for details).

The x axis represents different time points post invasion, and the y axis represents log_2 transformed $2^{-\Delta\Delta Ct}$ values (see appendix 4 for details). The horizontal solid blue line corresponds to the *PfPM2* mRNA level of 3D7 trophozoites (24h post invasion), used as a control. Black arrows indicate significant differences in *PfPM2* mRNA level (* p<0.05-0.011, ** p<0.01-0.0011 and *** p<0.001) between the 2 strains at each time point.

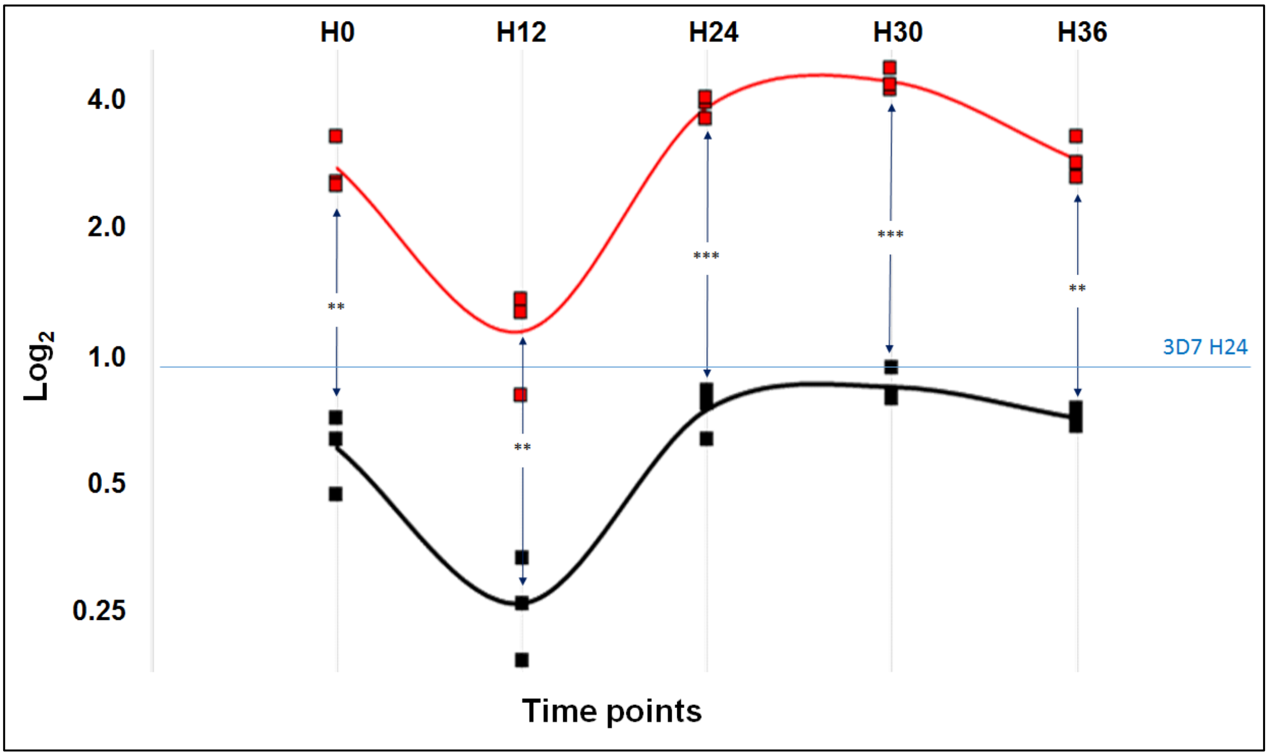

Panel B. PfPM2 expression in *Plasmodium falciparum* synchronized trophozoite-stage of piperaquine-resistant (6408, PSA survival rate=58.7%, multicopy *PfPM2*) and piperaquine-sensitive parasite lines (6267, PSA survival rate=0.5%, single copy *PfPM2*) detected by western immunoblot.

The piperaquine-resistant parasite line 6408 has higher PfPM2 protein levels than the sensitive line 6267. Synchronized trophozoite-stage cultures (24-30 hours post invasion) were probed with anti-PfPM2 (gift of Daniel Goldberg) and anti-beta-actin (NovusBio) antibodies. The resistant line has approximately twice as much PfPM2 as the sensitive line.

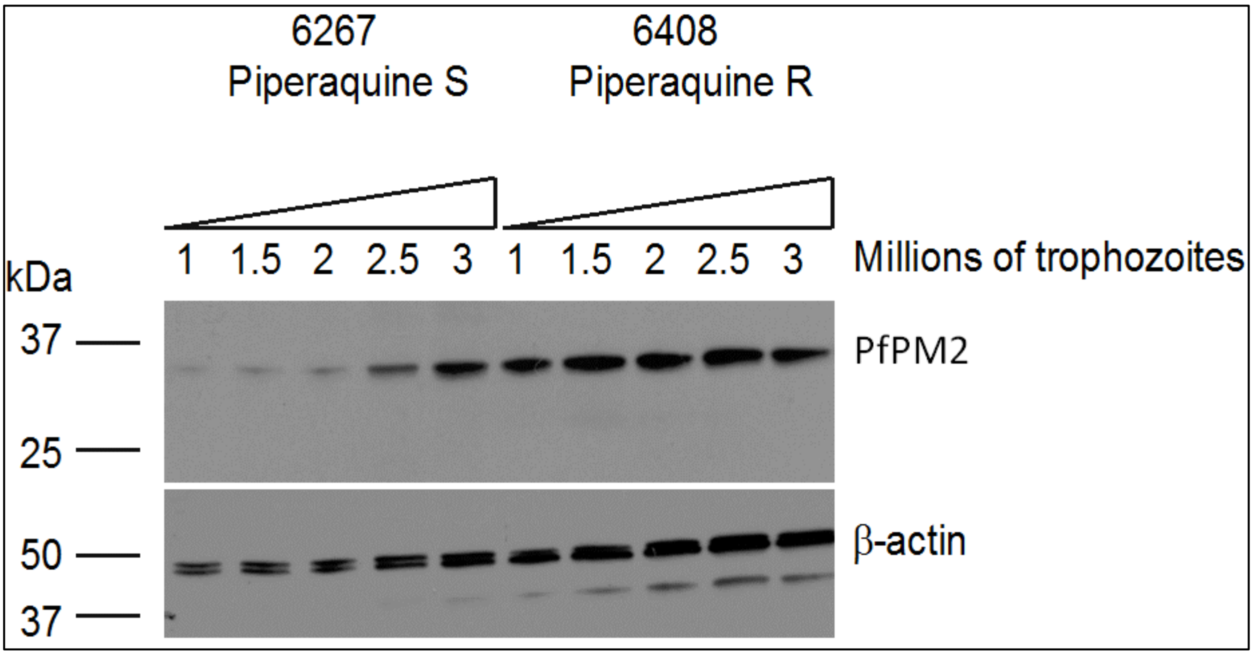

Appendix 8: Spatio-temporal increase in frequency of parasites with multicopy *PfPM2* in Cambodia from 2002 to 2015.

Panel A. Increase in frequency of parasites with multicopy *PfPM2* in Pailin (red histogram) and in Rattanakiri (black histogram) between 2002 and 2015. On the x-axis, sample sizes are given by site and year.

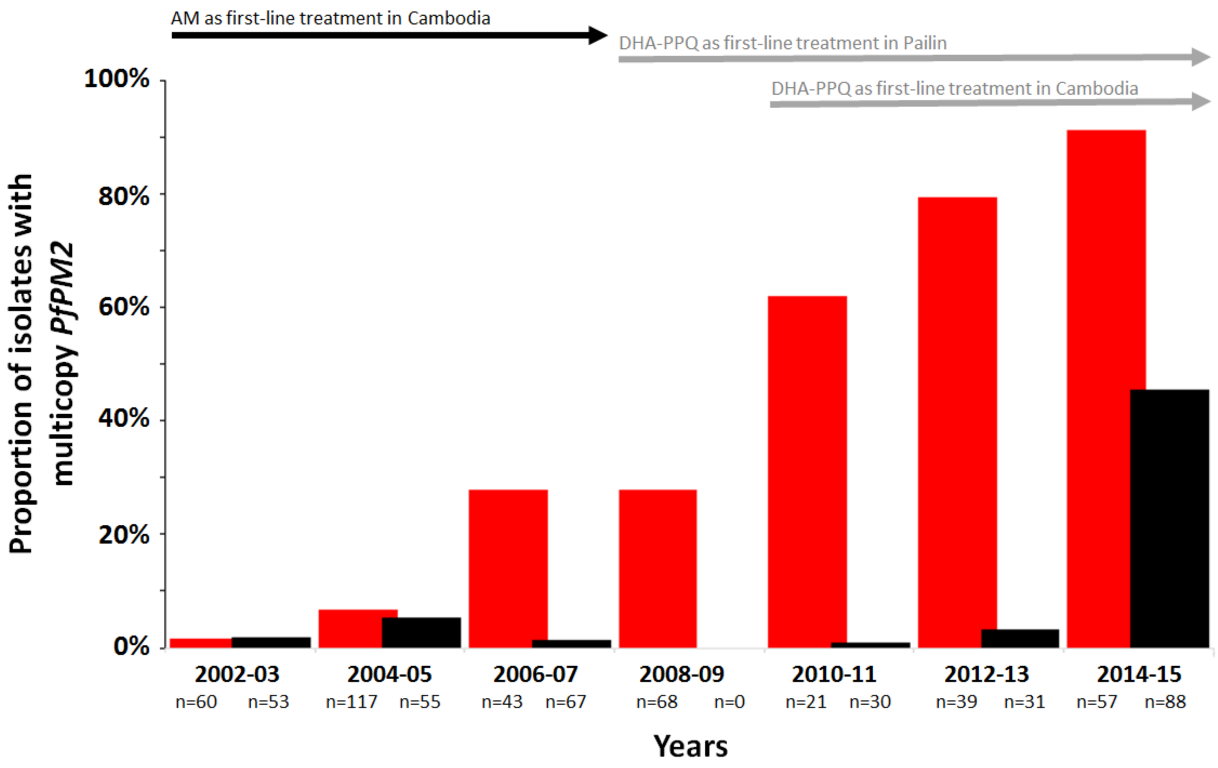

Panel B. Dynamic of the spread of multicopy *PfPM2* parasites in 10 Cambodian provinces from 2002 to 2015.

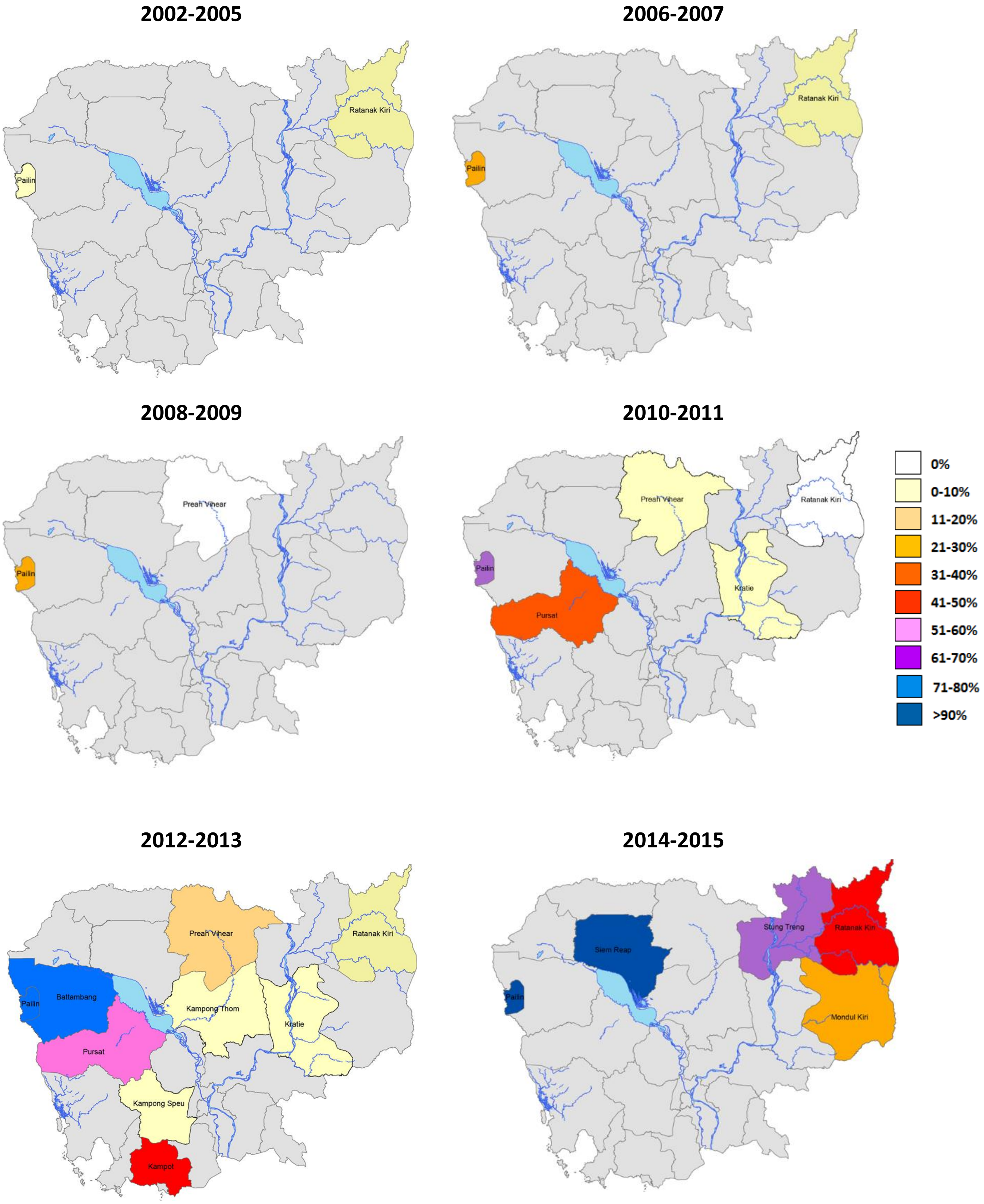

Legend: Color-codes are the proportion of parasites with multicopy *PfPM2*

Period (year)	Province	No isolates studied*	% isolates with multicopy *PfPM2*
**2002-2003**	Pailin	60	1.7
	Rattanakiri	53	1.9
**2004-2005**	Pailin	117	6.8
	Rattanakiri	55	5.4
**2006-2007**	Pailin	43	27.9
	Rattanakiri	67	1.5
**2008-2009**	Pailin	68	27.9
	Preah Vihear	30	0,0
**2010-2011**	Pailin	21	61.9
	Rattanakiri	30	0,0
	Pursat	73	38,4
	Kratié	51	2,0
	Preah Vihear	34	8,8
**2012-2013**	Pailin	39	79.5
	Rattanakiri	31	3.22
	Pursat	23	56.5
	Battambang	39	79.5
	Kampong Speu	22	4.5
	Kampo???ng Thom	38	7.9
	Kampot	17	47.1
	Kratié	22	4.5
	Preah Vihear	16	18.7
**2014-2015**	Pailin	57	91.2
	Rattanakiri	88	45.5
	Mondulkiri	55	25.4
	Siemreap	57	91.2
	Stungtreng	46	63.0

* Details regarding the samples collected from 2002 to 2008 are given in Ariey *et al.*, (Nature, 505, 50-55, 2014, A molecular marker of artemisinin-resistant *Plasmodium falciparum* malaria. (reference 1 of the main text).

Appendix 9:

Panel A. Correlation between the proportion of parasites with multicopy *PfPM2* and DHA-PPQ treatment failure rates recorded in 12 sites across Cambodia from 2009 to 2015.

Results from each clinical study (site and year) are represented by a colored dot. The position of the dot corresponds to the proportion of parasites with multicopy *PfPM2* (x axis) and the DHA-PPQ treatment failure rate (y axis). The color code refers to the proportion of K13 mutant parasites in each site by year.

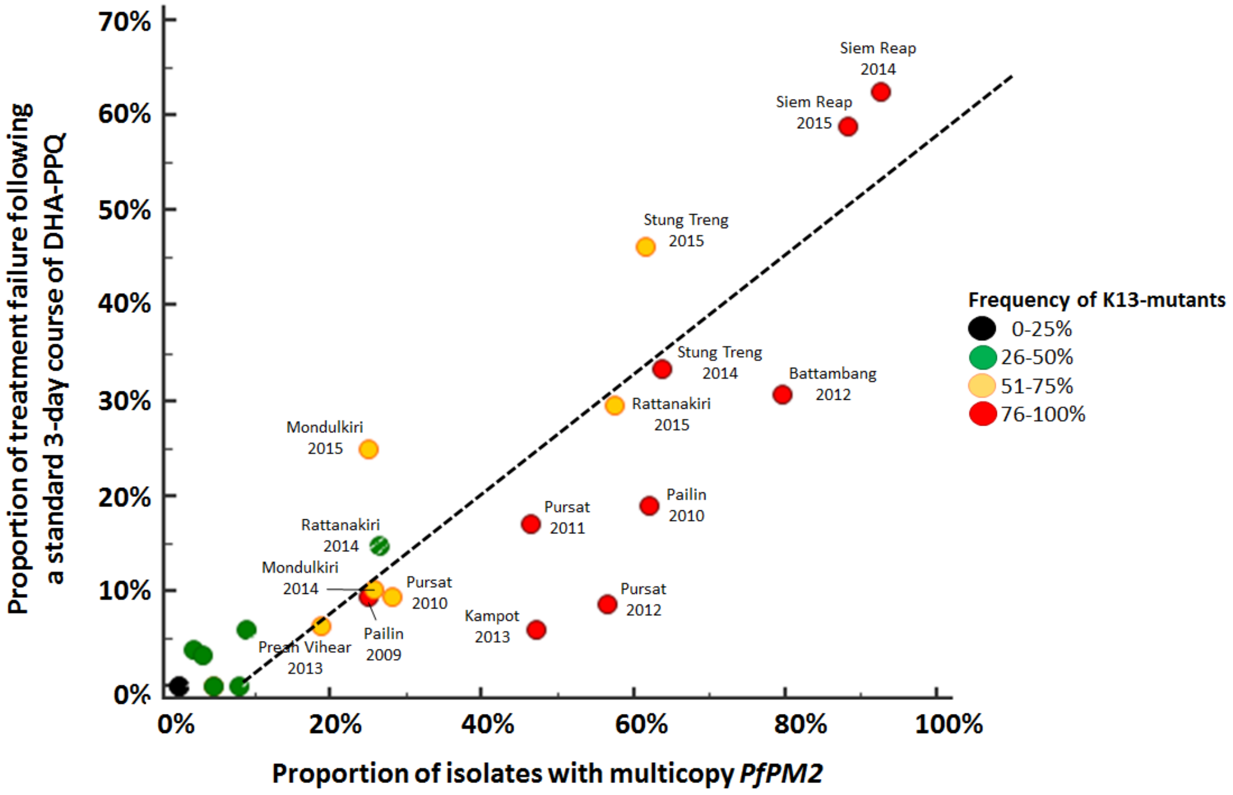

Panel B. Cox regression model: association of *PfPM2* copy number, sampling sites and treatment response.

Variables	coef	exp(coef)	se(coef)	z	p value
Kampong Speu	$-1.57.10^{1}$	$1.49.10^{-7}$	$2.95.10^{3}$	-0.01	0.9957
Kampong Thom	$-1.59.10^{1}$	$1.21.10^{-7}$	$2.08.10^{3}$	-0.01	0.9939
Kampot	-1.32	$2.68.10^{-1}$	1.04	-1.27	0.2059
Kratie	$1.30.10^{-2}$	1.01	$7.94.10^{-1}$	0.02	0.9870
Mondulkiri	$3.37.10^{-1}$	1.40	$4.58.10^{-1}$	0.73	0.4626
Pailin	$-8.79.10^{-2}$	$9.16.10^{-1}$	$4.76.10^{-1}$	-0.18	0.8536
Preah Vihear	$-2.54.10^{-1}$	$7.76.10^{-1}$	$6.55.10^{-1}$	-0.39	0.6988
Pursat	$-3.61.10^{-1}$	$6.97.10^{-1}$	$4.09.10^{-1}$	-0.88	0.3771
Rattanakiri	$4.33.10^{-1}$	1.54	$3.62.10^{-1}$	1.20	0.2311
Siem Reap	$9.18.10^{-1}$	2.51	$3.35.10^{-1}$	2.74	0.0061
Stung Treng	$5.77.10^{-1}$	1.78	$3.78.10^{-1}$	1.53	0.1268
PfPM2 amplification	3.44	$3.12.10^{1}$	$4.17.10^{-1}$	8.26	$<2.10^{-16}$

Likelihood ratio test=267 on 12 df, p=0

n= 725, number of events= 119

Appendix 10:

Panel A. Over-time trends of the proportions of isolates with different genetic background (K13 wild-type/*PfPM2* single copy, K13 mutant/*PfPM2* single copy, K13 mutant/*PfPM2* multicopy and K13 wild-type/*PfPM2* multicopy) observed in Cambodia from 2009 to 2015.

The color code is the same as in Figure 4B: K13 wild-type/*PfPM2* single copy (black), K13 wild-type/*PfPM2* multicopy (light purple), K13 mutant/*PfPM2* single copy (blue) and K13 mutant/ *PfPM2* multicopy (red).

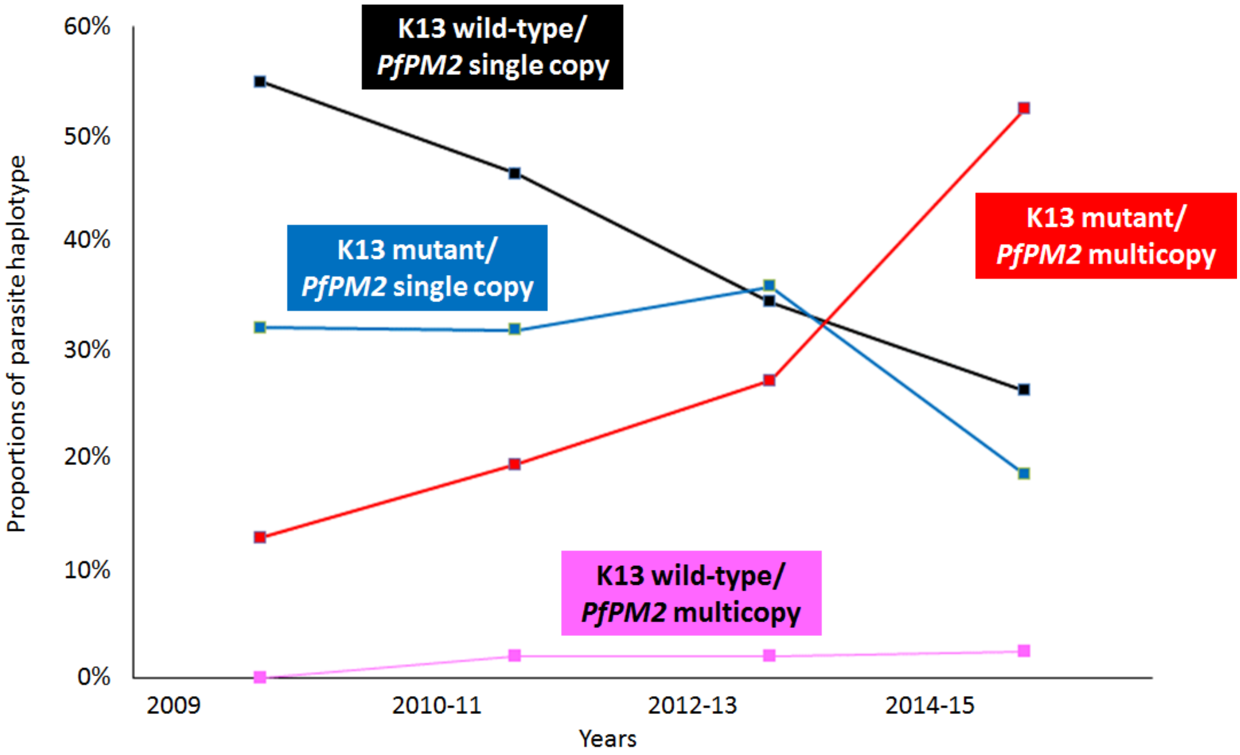

Panel B. Proposed scenario of the stepwise selection process for the emergence DHA-PPQ resistant parasites in Cambodia.

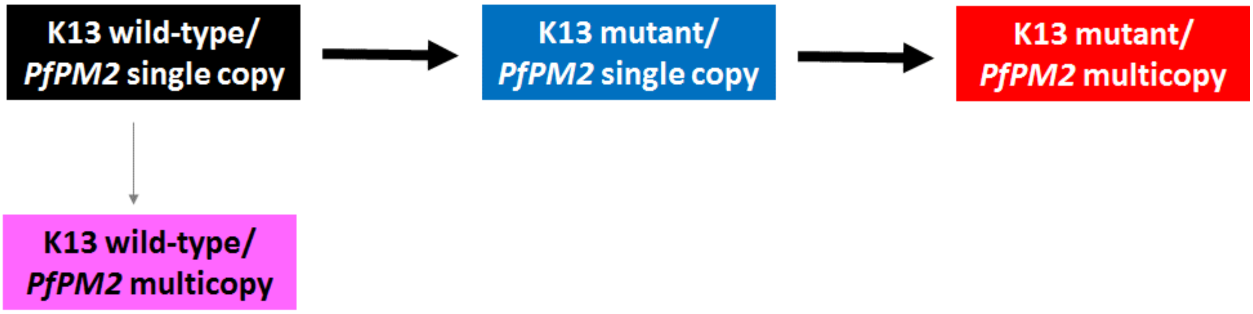

The thickness of the arrow is proportional to the probability of the selection process.

Appendix 11. Hypothesis supporting the mechanisms of resistance of *P. falciparum* parasites to PPQ through the amplification of *PfPM2* and *PfPM3* genes, and the deamplification of *Pfmdr1* gene in Cambodia.

Panel A. PPQ-sensitive parasite. PPQ accumulates in the food digestive vacuole via its weak-base properties. PfMDR1 transporter might help concentrate PPQ into the digestive vacuole, explaining the selection against multicopy *Pfmdr1*. PPQ inhibits hemoglobin degradation leading to the disruption of amino acid production. Parasite death is provoked.

Panel B: PPQ-resistant parasite. Amplification of the *PfPM2* and *PfPM3* genes and increased production of the PfPM2 and PfPM3 proteases is proposed to compensate for the PPQ inhibition of hemoglobin catabolism, restoring normal globin-derived peptide levels used for amino acid production, adequate intracellular osmotic pressure and promoting parasite survival. PfCRT might play a possible role in the efflux of PPQ from the digestive vacuole in some PPQ-resistant parasites.

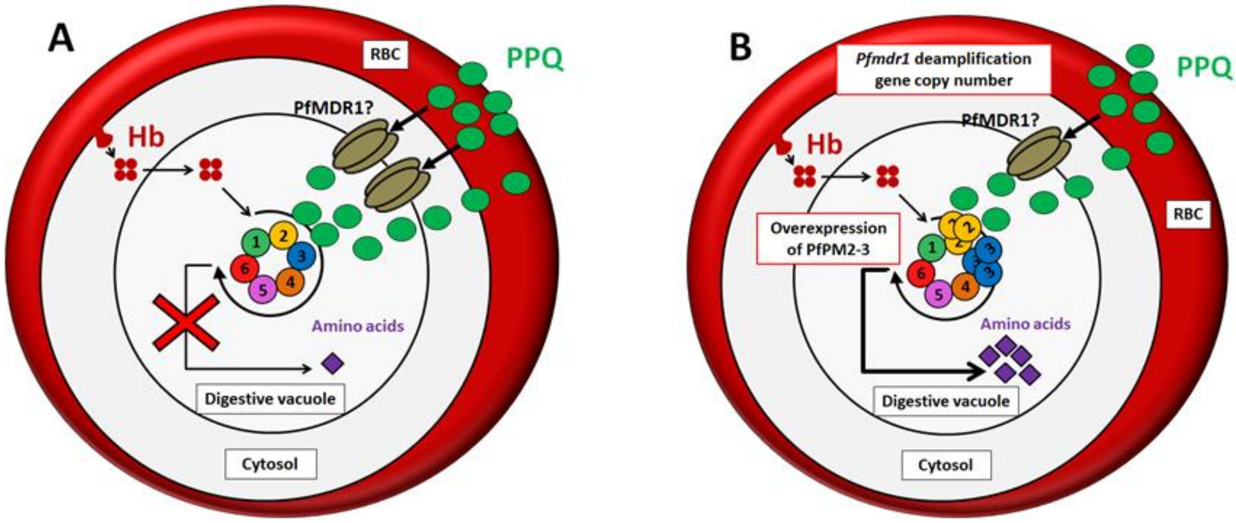

Hb: hemoglobin; 1: PfPM1; 2: PfPM2; 3: PfPM4; 4: PfFalcipain; 5: PfPM3; 6: PfFalcilysin.
